# *Iris domestica* (iso)flavone 7- and 3′-O-Glycosyltransferases Can Be Induced by CuCl_2_

**DOI:** 10.3389/fpls.2021.632557

**Published:** 2021-02-09

**Authors:** Xiang Zhang, Yan Zhu, Jun Ye, Ziyu Ye, Ruirui Zhu, Guoyong Xie, Yucheng Zhao, Minjian Qin

**Affiliations:** ^1^Department of Resources Science of Traditional Chinese Medicines, School of Traditional Chinese Pharmacy, China Pharmaceutical University, Nanjing, China; ^2^Key Laboratory of Modern Traditional Chinese Medicines (Ministry of Education), China Pharmaceutical University, Nanjing, China

**Keywords:** *Iris domestica (Belamcanda chinensis)* copper chloride, induce, (iso)flavones, transcriptome, UGT

## Abstract

In many plants, isoflavones are the main secondary metabolites that have various pharmacological activities, but the low water solubility of aglycones limits their usage. The O-glycosylation of (iso)flavones is a promising way to overcome this barrier. O-glycosyltransferases (UGTs) are key enzymes in the biosynthesis of (iso)flavonoid O-glycosides in plants. However, limited investigations on isoflavonoid O-UGTs have been reported, and they mainly focused on legumes. *Iris domestica (L.)* Goldblatt et Mabberley is a non-legume plant rich in various isoflavonoid glycosides. However, there are no reports regarding its glycosylation mechanism, despite the *I. domestica* transcriptome previously being annotated as having non-active isoflavone 7-O-UGTs. Our previous experiments indicated that isoflavonoid glycosides were induced by CuCl_2_ in *I. domestica* calli; therefore, we hypothesized that isoflavone O-UGTs may be induced by Cu^2+^. Thus, a comparative transcriptome analysis was performed using *I. domestica* seedlings treated with CuCl_2_, and eight new active BcUGTs were obtained. Biochemical analyses showed that most of the active BcUGTs had broad substrate spectra; however, substrates lacking 5-OH were rarely catalyzed. Real-time quantitative PCR results further indicated that the transcriptional levels of BcUGTs were remarkably induced by Cu^2+^. Our study increases the understanding of UGTs and isoflavone biosynthesis in non-legume plants.

## Introduction

Secondary metabolites derived from plants play very important roles in organisms' physiological activities, as well as human health. Isoflavonoid glycosides are a class of significant secondary metabolites mainly found in legume plants, which are associated with the interactions between legumes and both symbiotic and pathogenic microorganisms (Krämer et al., 1984; Graham et al., 1990; Udomsuk et al., 2010; Li et al., 2014; Clúa et al., 2018; Karre et al., 2019). However, the low water solubility of isoflavonoid aglycones is a barrier to the clinical applications. Glycosylation is an effective way to increase water solubility and bioavailability. For example, the apparent solubility of puerarin 7-O-glycoside is 18-fold that of puerarin (Jiang et al., 2008), and genistein 7-O-glycoside showed greater oral bioavailability than genistein (Kwon et al., 2007). O-glycosylation is the main glycosylation form, and O- UDP- sugar glycosyltransferases (O-UGTs) are responsible for the biosynthesis of various O-glycosides in plants. However, only several genes with isoflavone O-glycosylation ability have been reported, and moreover, they are mainly investigated in leguminous plants. For instance, eight *Medicago* UGTs have been identified as being active against various isoflavones and flavones using a functional genomics approach (Modolo et al., 2007). In soybean (*Glycine max*), GmUGT4 is highly specific for isoflavones, while GmUGT1 and GmUGT7 have broad substrate spectra (Funaki et al., 2015). *Pueraria lobate* UGT1 plays a role in isoflavone 7-O-glycosylation (Li et al., 2014), while PlUGT2 is a bifunctional isoflavone O-UGT responsible for both the 4′-O- and 4′,7-O-glycosylation of isoflavonoids (Wang et al., 2016). In non-legumes, isoflavone O-UGTs have only been reported in pomegranate and rice. UGT84A23, UGT84A24, and PgUGT95B2 cloned from pomegranate exhibit 7-O-glycosylation activity of genistein *in vitro* (Ono et al., 2016; Wilson et al., 2019). In the monocotyledonous plant rice, UGT709A4 and UGT706D1 could use isoflavones as substrates *in vitro*, and produce isoflavonoid 7-O-glycosides (Ko et al., 2008).

*Iris domestica* (L.) Goldblatt et Mabberley [syn. *Belamcanda chinensis* (L.) DC] forms a traditional Chinese medicine and isoflavones are the main active ingredients. Previous *in vitro* investigations showed that the isoflavones in rhizomes have a number of biological activities, including anti-mutagenic, anti-inflammatory, anti-angiogenic and anti-tumor activities, as well as inhibiting lipid peroxidation and scavenging free radicals (Seidlová-Wuttke et al., 2004; Zhang et al., 2005; Kang et al., 2009; Wozniak et al., 2010; Wang et al., 2013; Xie et al., 2014). Among the active isoflavones, O-glycosylated compounds, such as tectoridin and iridin, are the main ingredients (Lee et al., 2011; Chen et al., 2014). This implies that some isoflavone O-UGT genes may be abundant in *I. domestica*. In addition, it is unusual that *I. domestica* accumulates isoflavonoid glycosides as a non-legume. Thus, it is important for us to explore the mechanisms of glycosylation in *I. domestica*. However, *I. domestica* is a monocotyledonous plant; therefore, it is very difficult to obtain the active BcUGTs using homology cloning strategies owing to their low homology levels. Transcriptomes have aided in the discovery of isoflavone-related-UGTs in *I. domestica*. However, after analyzing the transcriptomes of six *I. domestica* organs, we did not identify any active (iso)flavone genes (Tian et al., 2018).

Previously, we found that the isoflavonoid glycoside content in *I. domestica* calli was significantly induced by Cu^2+^ stress. Specifically, when the isoflavones contents of calli were monitored for 49 days, tectoridin and iridin increased up to 1.14 mg/g and 0.95 mg/g, respectively, at 42 days after a 1.2-mM CuCl_2_ treatment. However, no tectoridin or iridin was detected in the control group (Zhu et al., 2020). The same dynamic content variation was observed in the adventitious roots of *Iris germanica* after a CuCl_2_ treatment (Tomoyoshi et al., 2005). Thus, CuCl_2_ appears to promote the biosynthesis of isoflavone glycosides by inducing related gene expression. Consequently, in this study, we chose CuCl_2_ as the elicitor to induce the expression of (iso)flavone 7-O-UGT genes in *I. domestica*. Using RNA sequencing (RNA-seq) and differentially expressed gene analyses, several BcUGT complementary DNAs (cDNAs), which belonged to the UGT706L family, were obtained. Biochemical analyses showed that eight active BcUGTs shared similar coding DNA sequences, but they exhibited different catalytic activities against different substrates. For most substrates, 7-O-glycoside compounds were the main products. Interestingly, a new compound was catalyzed by BcUGT4 and BcUGT5, and catalysis by the BcUGTs was 5-OH dependent. In addition, real-time quantitative PCR was performed to predict the functions of BcUGTs in *I. domestica* growth, especially its mechanism in CuCl_2_-induced gene expression. Our study elucidated the mechanisms of isoflavonoid glycoside biosynthesis in a non-legume plant through Cu^2+^ induction. Furthermore, it offers the potential to engineer new compounds using synthetic biological strategies.

## Materials and Methods

### Plant Materials and Stress Treatment

Newly harvested seeds of *I. domestica* were collected from the Botanic Garden of China Pharmaceutical University, Nanjing, China (31°54′36.36 N, 118°54′38.42 E). The seeds were identified by Prof. Minjian Qin (China Pharmaceutical University, Nanjing, China). After being sown in nutrient soil mixed with perlite, the seeds were placed in an artificial climate box at an ambient temperature of 25°C under a 14-h/10-h photoperiod. After 45 days of culturing, all the seedlings were transferred into liquid 1/2 Murashige and Skoog medium (MS, Murashige and Skoog, 1962) for 48 h. Then, 1.5 mM CuCl_2_ was added to the medium, while nothing was added to the control group medium. The concentration of CuCl_2_ was established in accordance with to the results of our previous copper stress-induced experiment (Zhu et al., 2020). Roots were collected after a 24-h CuCl_2_ treatment for RNA-seq and gene expression analyses. Another induction experiment was carried out to rule out the effect of the chlorine ion in the induction, 48-day-old seedlings were transferred into liquid 1/2 MS for 48 h. Then 1.5 mM MgCl_2_, 1.5 mM CaCl_2_, 1.5 mM ZnCl_2_, and 1.5 mM CuCl_2_ was added to the medium respectively, while nothing was added to the control group medium. Roots were collected after a 24-h treatment for gene expression analyses. All the samples were collected in three independent biological replicates. Tissues from different organs (flower, fruit, leaf, rhizome, and root) were collected separately in three biological replicates from three independent plants, these samples were frozen immediately in liquid nitrogen after washing and maintained at −80°C until use.

### RNA Extraction and cDNA Library Preparation

Total RNAs were extracted using an RNA simple total RNA kit (TIANGEN, Beijing, China), and the roots were collected from the seedlings treated with CuCl_2_ and the control group. RNA degradation and contamination were examined using 1% agarose gels. RNA purity was checked using a NanoPhotometer® spectrophotometer (Implen, Carlsbad, CA, USA). The RNA concentration was measured using a Qubit® 2.0 Fluorometer (Life Technologies, Carlsbad, CA, USA). RNA integrity was assessed using the RNA Nano 6000 Assay Kit of the Agilent Bioanalyzer 2100 system (Agilent Technologies, Santa Clara, CA, USA). Sequencing libraries were generated using the NEB Next Ultra RNA Library Prep Kit for Illumina (New England Biolabs, Seattle, WA, USA).

### Illumina Sequencing, Data Filtering, Transcriptome Assembly, and Gene Functional Annotation

Six sequencing libraries of *I. domestica* were subjected to the Illumina Novaseq 6000 platform (Illumina, San Diego, CA, USA) to generate paired-end reads. After removing low-quality reads and reads containing adapters or poly-N, we obtained clean data that were then assembled into contigs using the Trinity assembler (Grabherr et al., 2011). A gene functional annotation was performed in seven public databases: NCBI non-redundant protein sequences, NCBI non-redundant nucleotide sequences, Pfam, euKaryotic Ortholog Groups/Clusters of Orthologous Groups of proteins, Swiss-Prot, Kyoto Encyclopedia of Genes and Genomes, and Gene Ontology.

### Screening of Candidate BcUGTs

Clean data were mapped back onto the assembled transcriptome. The read count for each gene was obtained from the mapping results using the RNA-seq by Expectation–Maximization (RSEM) software package (Li and Dewey, 2011). The expression level of each transcript in each sample was described in terms of fragments per kilobase of exon model per million mapped reads, which is a calculation of normalized read counts. A differential expression analysis of two samples was performed using the DESeq R package (Anders and Huber, 2010). Genes having an adjusted *p*-value < 0.005 and |log2 Foldchange| > 1 were considered as differentially expressed. To search for genes involved in the biosynthesis of isoflavone 7-O-glycosides in *I. domestica*, we established a local database using the transcriptome data, and then performed a local BLAST algorithm-based search to identify genes that shared similar conserved sequences with isoflavone 7-O-UGT genes of legume plants. To further determine the evolutionary relationships among these genes, the BcUGTs proteins were aligned with sequences of other plant (iso)flavone O-UGT genes having known functions using MEGA7 (https://www.megasoftware.net/). A phylogenetic tree was constructed using the neighbor-joining method, with a bootstrap of 1,000. The evolutionary distances were computed using the Poisson correction (Kumar et al., 2016).

### Functional Analysis of BcUGTs Candidates *in vitro*

The open reading frames of BcUGTs were cloned individually into the prokaryotic expression vector pCold-TF, which helps the proteins fold correctly owing to the expression of the coupled trigger factor (chaperone). Consequently, the efficient expression of soluble proteins that are difficult to deal with in other systems may be achieved. The primers used for the cloning are listed in Supplementary Table 1. All the BcUGT-pCold-TF constructs were transferred into *Escherichia coli* after sequencing. The recombinant strains were then inoculated into 100 mL Luria Broth liquid culture medium at 37°C and 220 rpm in an orbital shaker incubator. When the OD_600_ of the strains reached 0.6, 0.5 mM isopropyl 1-β-D thiogalactoside (IPTG) was added into the culture to induce the expression of recombinant proteins. This was followed by further incubation at 15°C for 24 h. The recombinant *E. coli* cells were harvested by centrifugation (10,000 g for 10 min at 4°C), resuspended with 50 mM phosphate-buffered saline (PBS; pH 8.0) and disrupted using a sonicator. The recombinant proteins were purified using Ni-NTA columns (Sangon, Shanghai, China). The purified proteins were subjected to SDS-PAGE in accordance with the method of Laemmli (1970), and the protein concentrations were determined using the Bradford method (Bradford, 1976).

### Enzyme Assays

The catalytic UGT reactions were performed in 200 μL. The reaction components included 2 mM uridine diphosphate glucose (UDPG; Sigma-Aldrich, Shanghai, China), 1 mM dithiothreitol, 50 mM PBS, 10 μg of the purified recombinant protein, and 100 μM (iso)flavonoid acceptors. The reactions were incubated for 1 h at 30°C and then 400 μL ethyl acetate was added to stop the reactions. Ethyl acetate was vaporized to dryness in the vacuum drying oven, and finally, the samples were dissolved in 100 μL methanol for HPLC analysis. Firstly, we calculated the amount of residual substrate, then the ratio of residual substrate in total initial substrate was obtained, and the ratio is the substrate conversion rate. For kinetic studies of BcUGTs, typical assays containing 2 mM UDPG, 1 mM dithiothreitol, 50 mM PBS, 2 μg purified recombinant protein, and varying concentrations of substrates were performed. The reactions were incubated for 10 min at 30°C, and the solutions subjected to HPLC were prepared as above. Kinetic parameters were determined by hyperbolic regression analyses using the Prism program (GraphPad, https://www.graphpad.com/). The reaction products were analyzed using an Agilent 1100 HPLC system on SB C18 columns (5 μm; 250 × 4.6 mm; Agilent Technologies) in which solvent A was 0.1% formic acid in water and solvent B was 100% acetonitrile. The substrates and products were eluted using a linear gradient of 30% B to 60% B in 15 min at a flow rate of 1 mL/min. The chromatograms were obtained with detections at 270 nm (for isoflavones), 280 nm (for flavanones), 340 nm (for flavones), and 360 nm (for flavonols).

### Homology Modeling and Auto-Docking Analysis

We chose BcUGT4 to generate a protein model using SWISS-MODEL (https://swissmodel.expasy.org/, Biasini et al., 2014), the templates were searched using BLAST (Camacho et al., 2009) and HHblits (Steinegger et al., 2019). We used GMQE (Global Model Quality Estimation) to estimate the template quality, GMQE score is expressed as a number between 0 and 1, higher numbers indicate higher reliability. The quality of protein model was evaluated *via* GMQE and QMEAN (Benkert et al., 2008). Docking of naringenin and liquiritigenin to BcUGT4 model was performed using AutoDock Tools (Trott and Olson, 2010, version 1.5.6). We used the default parameters in AutoDock Tools to prepare the ligands and macromolecule, then ran AutoDock to acquire the docking structures. Finally, we used PyMOL to present the docking results (https://pymol.org/2/).

### Transcriptional Aanalysis

The RNAs of seedling roots treated with MgCl_2_, CaCl_2_, ZnCl_2_, and CuCl_2_ were isolated as above. Then, different organs of *I. domestica* and different parts of rhizomes were extracted to detect the expression levels of BcUGTs, and samples were all collected in three independent biological replicates. The quality and the quantity of total RNAs were determined using a NanoDrop Spectrophotometer (Eppendorf, Germany) and 1% agarose gels. The first-strand cDNA for real-time PCR was synthesized using HiScript II Q RT SuperMix for qPCR (Vazyme, Nanjing, China). The PCR reaction was carried out in a total volume of 20 μL containing ChamQ SYBR qPCR Master Mix (Vazyme) on an ABI StepOne plus Real-time PCR system (Applied Biosystem, USA), with cycling parameters 95°C for 30 s and 40 cycles of 95°C for 5 s and 60°C for 30 s. The relative quantification method (ΔΔ^−*CT*^) was used to calculate the expression level of each unigene (Livak and Schmittgen, 2001). The specificity of primers was tested using a melting curve program. The slope of the relative standard curve was calculated to ensure the amplification efficiency. The primers used for qPCR are listed in Supplementary Table 2, and each gene has three biological replications and three technical replications.

## Results

### *De novo* Sequencing and Functional Annotation of Unigenes

Our previous study showed that calli of *I. domestica* treated with CuCl_2_ accumulated 2.09 mg/g of tectoridin and iridin, while no isoflavonoid glycosides were detected in the control group. Thus, 45-day-old *I. domestica* seedling roots treated with CuCl_2_ were used for Illumina sequencing after the RNA quality met specific criteria (Supplementary Figure 1). The adapter, unknown and low-quality reads were removed to obtain clean reads. In total, 25.03 and 25.57 Gb clean bases from the control and treated groups, respectively, were obtained and used for transcript assembly (Supplementary Table 3). A total of 73,132 unigenes were annotated in the seven referenced public databases, with 24.6% annotated unigenes being homologous to *Asparagus officinalis*, followed by *Elaeis guineensi* and *Phoenix dactylifera*. Gene Ontology and Kyoto Encyclopedia of Genes and Genomes annotations assigned the unigenes to different metabolic pathways. This was the basis for the preliminarily functional predictions for these unigenes (Supplementary Figures 2, 3). These results provided a good foundation for the screening of candidate BcUGTs.

### Selection of Candidate BcUGTs Induced by CuCl_2_

**Figure 1:**
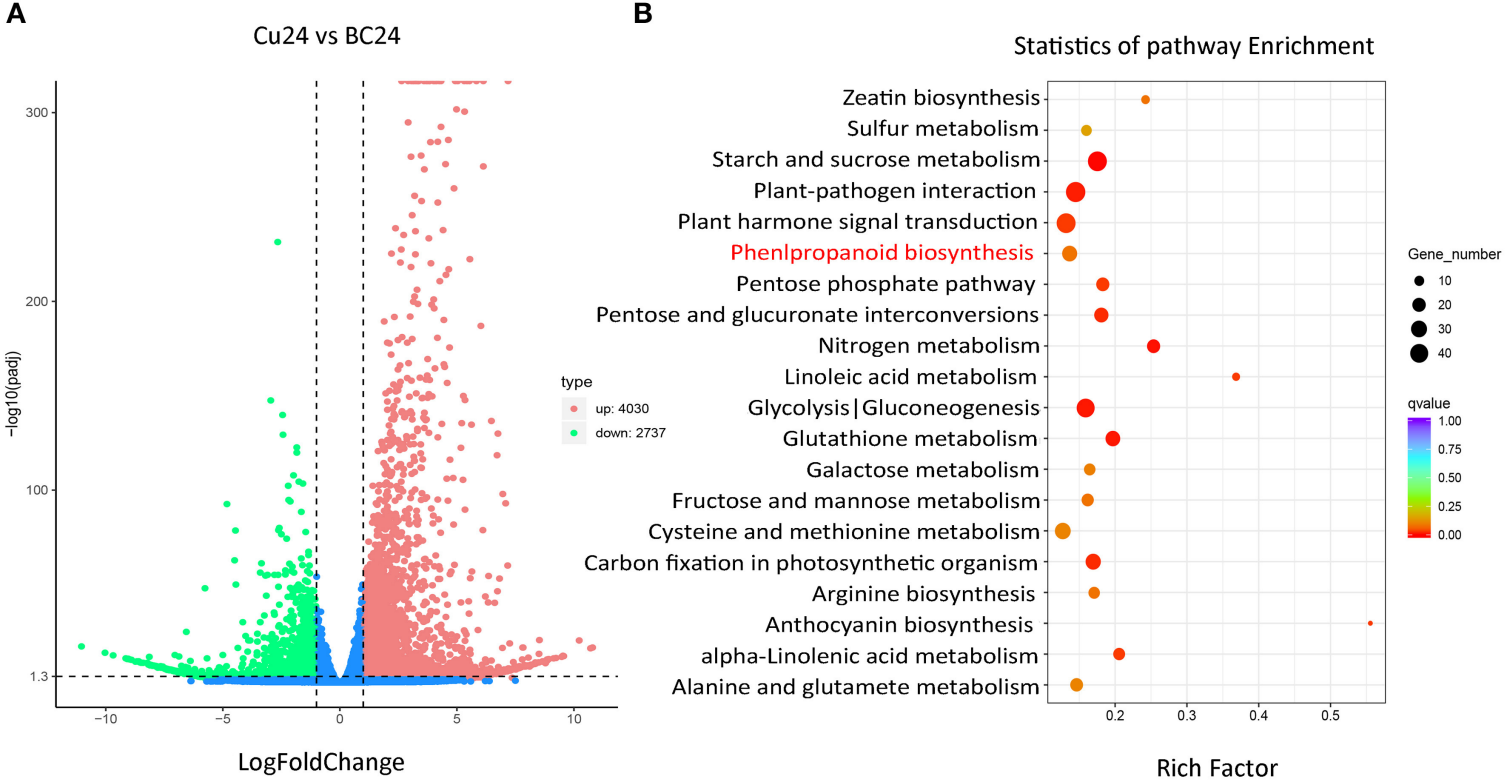
Differential expression analysis of the *I. domestica* transcriptome induced by CuCl_2_. **(A)** Volcano plots of differentially expressed genes; **(B)** Statistics of enrichment by KEGG. Cu24, the root samples treated by CuCl_2_ for 24 h; BC24, the control groups. The control groups and the experimental groups are both in three independent biological replicates.

**Figure 2:**
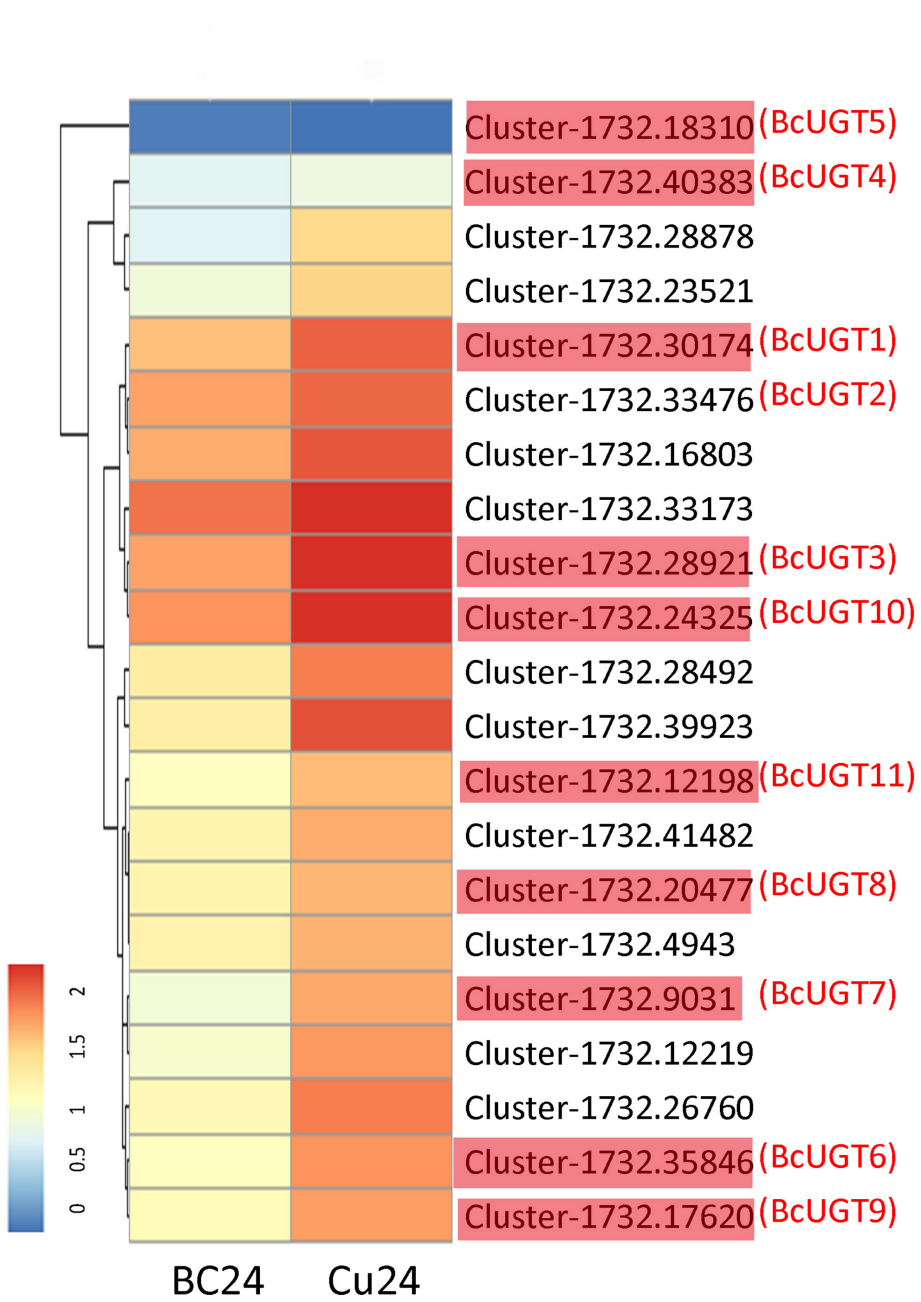
Analysis of differentially expressed unigenes from *I. domestica*. Hierarchical clustering and corresponding heatmaps of the differentially expressed (iso)flavone glycosyltransferases across all pairwise library comparisons. The clusters in red (BcUGT1–11) were selected to verify the activity against flavones *in vitro*. Cu24, the root samples treated by CuCl_2_ for 24 h; BC24, the control groups. The control groups and the experimental groups are both in three independent biological replicates.

Genes involved in the same biosynthetic route are temporally and spatially induced by effective treatments, which indicates that the gene functions may be characterized by a differential expression analysis. Using the standard criteria of an adjusted *p*-value < 0.005 and |log2 Foldchange| > 1, we obtained 6,767 differentially expressed genes. Among them, 4,030 were up-regulated in *I. domestica* roots treated with CuCl_2_, and many unigenes were enriched in phenylpropanoid biosynthesis (Figure 1). Then, 19 candidate unigenes were selected from the up-regulated unigenes annotated as O-UGTs. To fully screen for possible unigenes involved in isoflavonoid glycoside biosynthesis, two putative UGTs (Cluster-1732.40383 and Cluster-1732.18310) were identified using a local BLAST algorithm-based search, and they shared similar conserved sequences with GmUGTs. The expression levels of these 21 candidate genes were up-regulated after CuCl_2_ treatments, except Cluster-1732.18310, and the expression levels of most of the selected genes increased remarkably, having log2 Foldchanges > 2 (Figure 2). The sequences of the 21 genes were used as the query in a BLAST algorithm-based search of National Center for Biotechnology Information (NCBI) databases to predict their gene families and possible functions. We finally selected 11 BcUGTs putatively identified as (iso)flavone-O-UGTs to determine whether they were capable of transferring UDPG to (iso)flavone aglycones (Figure 2).

### Functional Characterization of Active BcUGTs *in vitro*

For *in vitro* biochemical assays, the open reading frame sequences of the selected 11 BcUGTs were all cloned independently into the prokaryotic expression vector pCold-TF and expressed in *E. coli* cells. The recombinant proteins purified with Ni-NTA columns showed strong and single protein bands at 105 kDa by SDS-PAGE (Supplementary Figure 4). The deduced molecular weights of the BcUGTs were ~53 kDa, with the fusion protein having an additional soluble label and His-tag of ~52 kDa. The protein bands identified by SDS-PAGE indicate that these soluble proteins may be used to determine the enzymes' activity levels and kinetic properties. The functional BcUGT assays were performed against the different (iso)flavone substrates: naringenin, apigenin, kaempferol, irigenin and tectorigenin. BcUGT1–8 showed activities toward (iso)flavones, while Cluster-1732.24325, Cluster-1732.17620 and Cluster-1732.12198 did not transfer a glycosyl group onto (iso)flavones, although they were annotated as (iso)flavone UGTs. BcUGT1–8, except BcUGT8, shared very high deduced amino acid sequence similarities, ranging from 81.7 to 97.3% (Supplementary Table 4), all of them were submitted to the UGT Nomenclature Committee and designated as UGT706L family genes.

**Figure 3:**
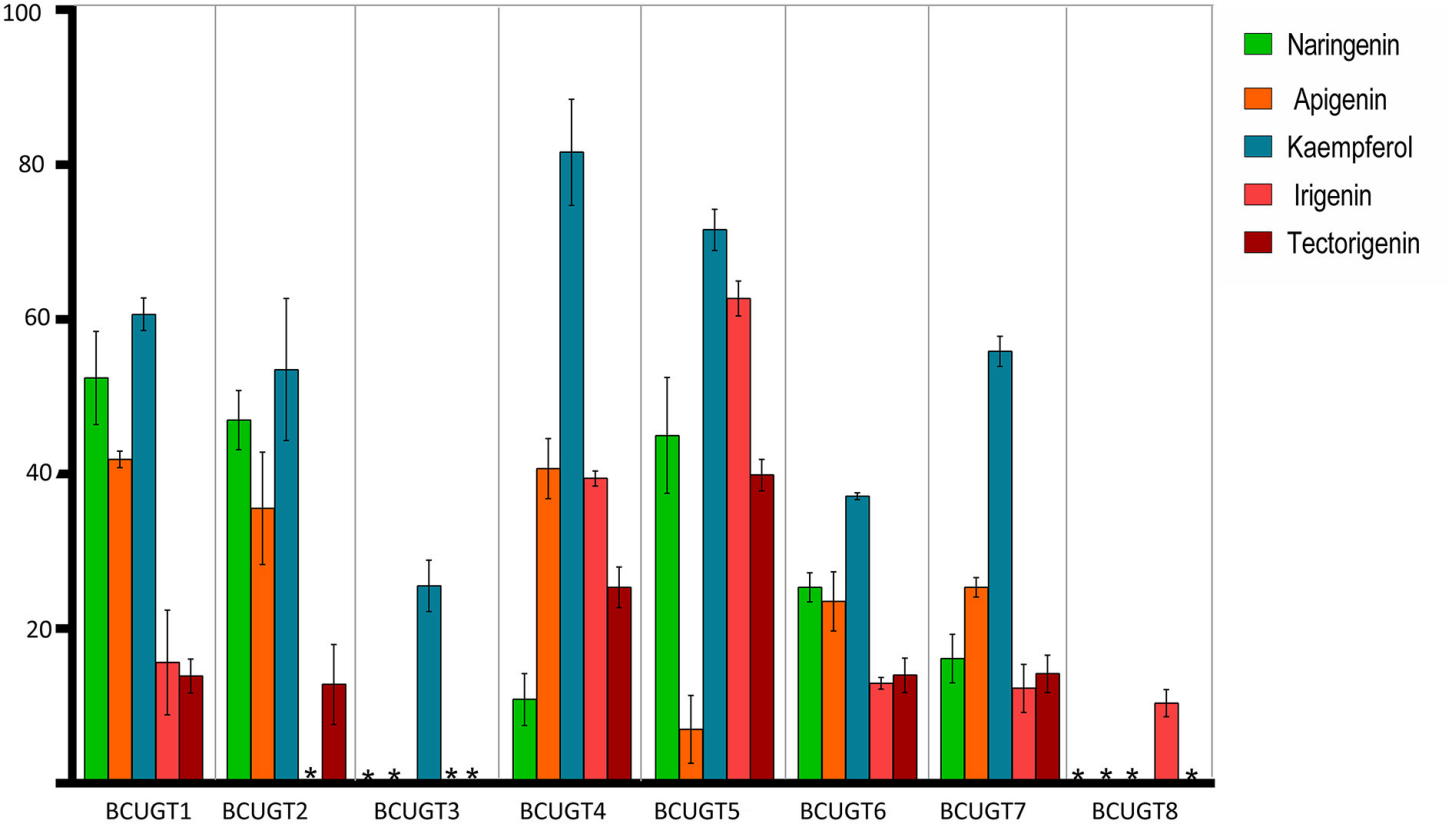
Substrate conversion rates of BcUGTs with flavonoids. The reactions were performed at pH 8.0 in 30°C for 1 h with 100 μM each substrate, naringenin, apigenin, kaempferol, irigenin, and tectorigenin. The values were calculated as the averages of three replicates of each reaction. Asterisks, activity not detected.

The substrate conversion rates were determined using the flavones: naringenin, liquiritigenin, apigenin, baicalein, luteolin, quercetin, kaempferol, irigenin, tectorigenin, genistein, daidzein, and 6,7,4′-trihydroxyisoflavone (Supplementary Figure 5). BcUGT1, −2 and −4–7 displayed broad activities toward various (iso)flavone substrates, with flavonols being the most preferred (Supplementary Figure 6). However, for different types of substrates, BcUGTs exhibited different catalytic efficiencies. As shown in Figure 3, BcUGT1 exhibited similar catalytic activities toward different sugar acceptors, as did BcUGT2, while BcUGT4 was analogous to BcUGT5. Among these substrates, BcUGT3 only accepted luteolin and kaempferol as sugar acceptors, while BcUGT8 only exhibited relatively low activities toward luteolin and irigenin. The other flavonoids were all inert substrates for these enzymes (Table 1). The conversion rates of BcUGT4 and BcUGT5 for irigenin and tectorigenin were greater than other BcUGTs, by 2–5 times (Table 2).

**Table 1:** Substrate conversion rates of BcUGTs with various flavonoids.

**Substrate**	**Conversion rate %**							
	**BcUGT1**	**BcUGT2**	**BcUGT3**	**BcUGT4**	**BcUGT5**	**BcUGT6**	**BcUGT7**	**BcUGT8**
**Flavanone**								
Naringenin	52.4 ± 6.0	47.0 ± 3.8	0.0	47.0 ± 3.8	45.0 ± 7.5	25.3 ± 1.9	16.1 ± 3.1	0.0
Liquiritigenin	20.6 ± 5.0	11.2 ± 3.4	0.0	16.1 ± 3.1	7.8 ± 2.4	16.2 ± 1.1	11.3 ± 1.5	0.0
**Flavone**								
Apigenin	41.9 ± 1.1	35.6 ± 7.3	0.0	40.7 ± 3.9	7.0 ± 4.4	25.5 ± 3.8	25.4 ± 1.2	0.0
Baicalein	42.5 ± 3.5	41.7 ± 1.5	0.0	9.1 ± 3.9	7.9 ± 6.4	15.9 ± 3.7	25.9 ± 2.1	0.0
Luteolin	29.9 ± 3.5	28.0 ± 4.0	13.7 ± 3.9	44.8 ± 9.2	21.3 ± 8.2	18.6 ± 3.7	35.2 ± 1.7	9.7 ± 2.4
**Flavonol**								
Quercetin	53.6 ± 7.5	62.5 ± 4.3	0.0	39.2 ± 7.1	26.3 ± 4.2	12.2 ± 0.8	12.4 ± 1.8	0.0
Kaempferol	60.6 ± 2.1	53.5 ± 9.2	25.5 ± 3.3	81.6 ± 6.9	71.6 ± 2.6	37.1 ± 0.5	55.8 ± 1.9	0.0
**Isoflavone**								
Tectorigenin	13.9 ± 2.2	12.8 ± 5.2	0.0	25.3 ± 2.7	39.9 ± 2.0	14.0 ± 2.2	14.2 ± 2.4	0.0
Irigenin	15.6 ± 6.8	0.0	0.0	39.4 ± 1.0	62.7 ± 2.3	12.9 ± 0.8	12.3 ± 3.1	10.4 ± 1.8
Genistein	11.1 ± 2.8	16.2 ± 7.6	0.0	39.9 ± 1.0	13.5 ± 1.9	24.8 ± 0.2	17.5 ± 2.3	0.0
Daidzein	0.0	0.0	0.0	0.0	0.0	0.0	0.0	0.0
6,7,4'-Trihydroxy-isoflavone	0.0	0.0	0.0	0.0	0.0	0.0	0.0	0.0

*Reactions of BcUGTs with 100 μM substrate at pH 8.0 and 30°C for 1 h. Data are the means ± SDs of one experiment with three technical replicates*.

**Table 2:** Kinetic parameters of BcUGTs with tectorigenin and irigenin.

**Substrate**	**kat(s^**−1**^)**	**km(mM)**	**kcat/km(M^**−1**^s^**−1**^)**
**BcUGT1**			
Tectorigenin	57.29 ± 4.94	0.592 ± 0.049	9.68 × 10^4^
Irigenin	58.88 ± 3.24	0.210 ± 0.013	2.80 × 10^5^
**BcUGT2**			
Tectorigenin	70.10 ± 9.86	0.392 ± 0.007	1.79 × 10^5^
Irigenin	–	–	–
**BcUGT4**			
Tectorigenin	155.18 ± 12.26	0.115 ± 0.023	1.35 × 10^6^
Irigenin	57.23 ± 18.21	0.190 ± 0.007	3.01 × 10^5^
**BcUGT5**			
Tectorigenin	116.03 ± 6.98	0.201 ± 0.015	5.77 × 10^5^
Irigenin	296.52 ± 11.8	0.121 ± 0.009	2.45 × 10^6^
**BcUGT7**			
Tectorigenin	15.86 ± 7.53	1.269 ± 0.009	1.98 × 10^4^
Irigenin	17.73 ± 9.89	0.894 ± 0.011	1.98 × 10^4^
**BcUGT8**			
Tectorigenin	–	–	—
Irigenin	11.97 ± 1.31	1.423 ± 0.021	8.41 × 10^3^

*Kinetic parameters were determined at pH 8.0 in 30°C with UDP-glucose, as described in the Materials and methods section. Data are the means ± SDs of one experiment with three technical replicates*.

**Figure 4:**
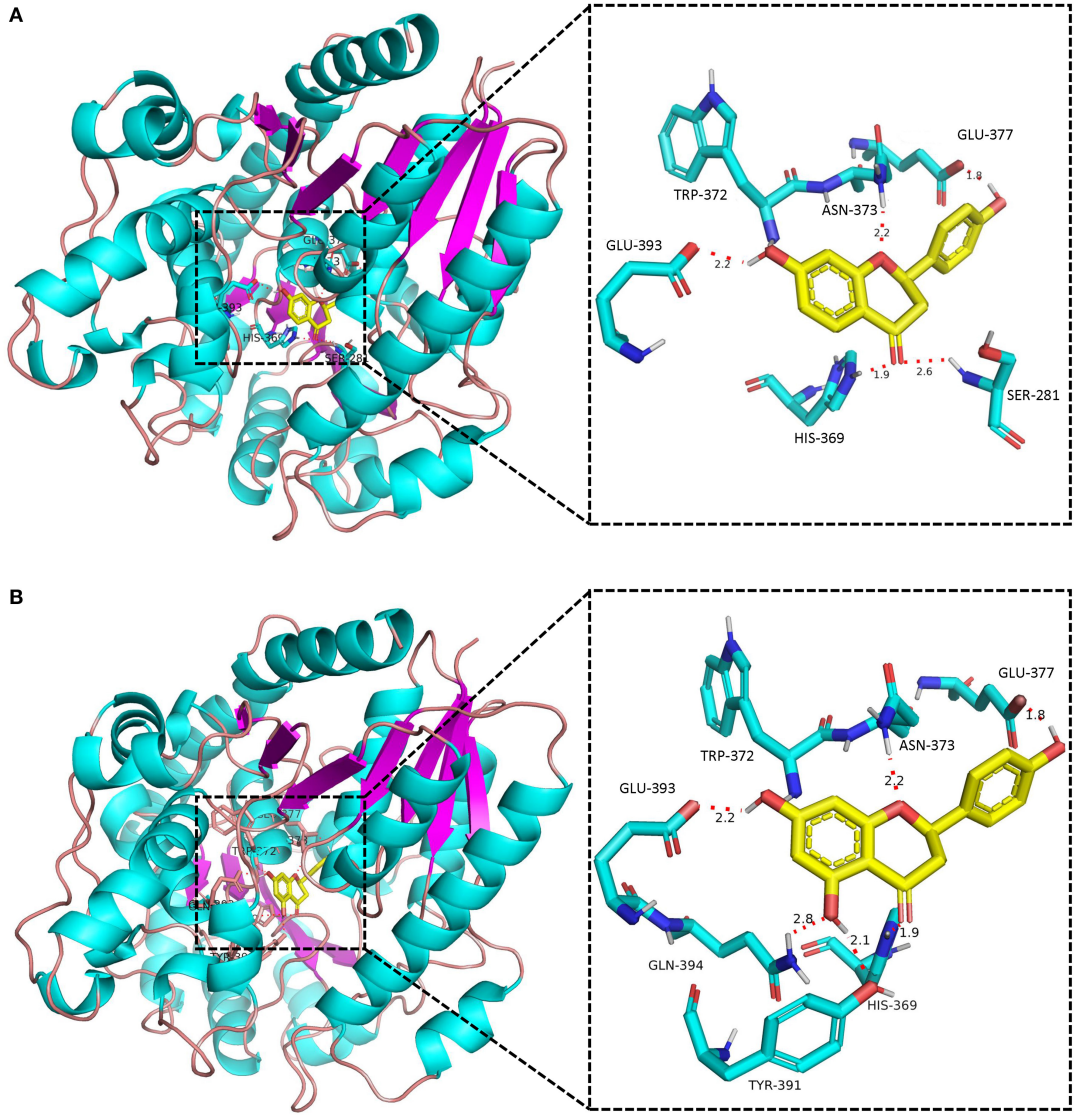
Docking model of BcUGT4 with liquiritigenin and naringenin. The protein model of BcUGT4 was established using SWISS-MODEL. The enlarged region shows polar interactions of substrates binding with BcUGT4 residues. **(A)** Autodocking result of BcUGT4 with liquiritigenin; **(B)** Autodocking result of BcUGT4 with naringenin.

None of the BcUGTs displayed any activity when daidzein and 6,7,4′-trihydroxyisoflavone were used as substrates. These isoflavones are rich in soybean, and their chemical structures lack 5-OH (Table 1). BcUGT1 and BcUGT5 converted 2.5-fold and 5.6-fold more naringenin, respectively, to their 7-O-glycosides compared with liquiritigenin. In addition, for daidzein and 6,7,4′-trihydroxy-isoflavone, none of the BcUGTs showed any activity. Thus, the existence of 5-hydroxy of (iso)flavone aglycones was very important for catalysis by the BcUGTs. In this study, BcUGT4 was chosen to investigate the critical role of the 5-hydroxy in protein binding with substrates, we identified the top 50 templates according to the coverage, identity, and GMQE. A newly reported TcCGT1 (PDB) crystal structure was used as the template for homologous molecular modeling in SWISS-MODEL, the GMQE score of TcCGT was 0.75. A C-glycosyltransferase found in *Trollius chinensis*, TcCGT1, regio-specifically produces 8-C-glycosylation flavones with broad substrate spectra, and site-directed mutagenesis at just two residues switched C- to O-glycosylation (He et al., 2019). The GMQE of the model was 0.72, and the GMEAN was −2.59, GMEAN scores > −4 is an indication of models with high quality. Afterward, we modeled the structure of BcUGT4 docking with liquiritigenin and naringenin (Figure 4). The spacious binding pocket explained the broad substrate spectra of BcUGT4, with seven residues interacting with naringenin through hydrogen bonds having 1.8–2.8 Å distances. The residues GLN-394 and TYR-391 interacted with naringenin C-5-OH (2.8 Å, 2.1 Å), but for liquiritigenin, five residues, HIS-369, GLU-393, TRP-372, ASN-373, and GLU-377, were discovered around the ligand and they were also identified in the naringenin docking.

### Structural Analysis of the New Compound

**Figure 5:**
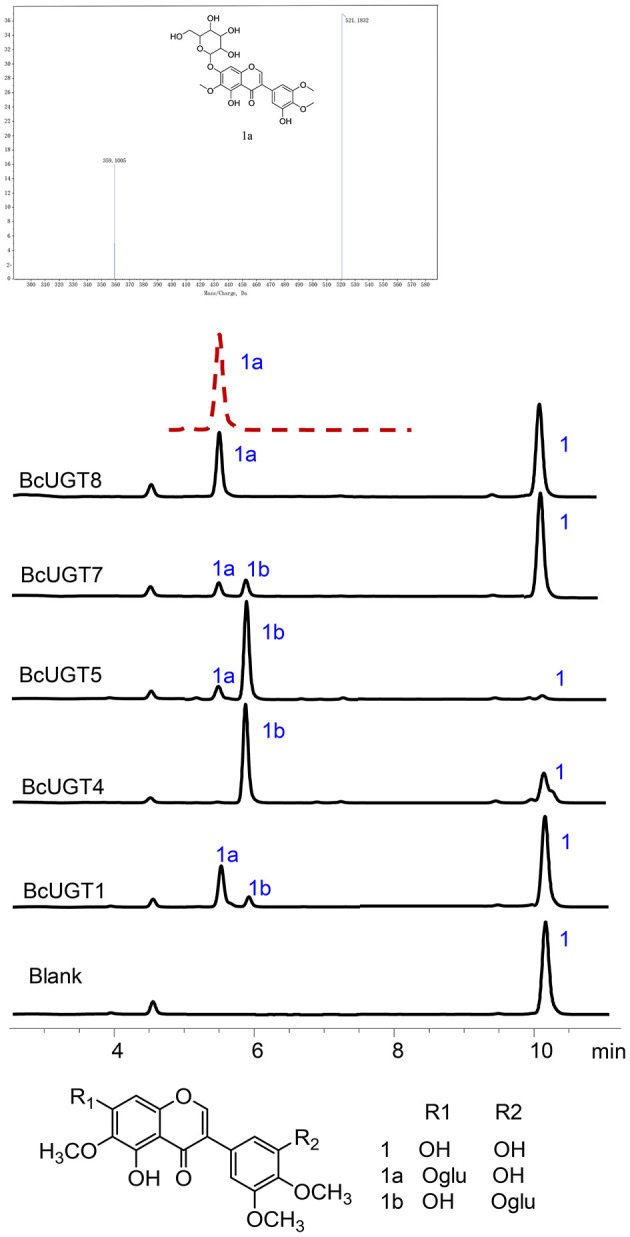
HPLC analyses of BcUGT reactions with irigenin and mass spectrum of 1a. A reaction mixture of irigenin and UDP-glucose with BcUGTs. 1, Irigenin; 1a, Irigenin-7-O-glycoside; 1b, Irigenin-3′-O-glycoside; red dashed line, Authentic irigenin-7-O-glycoside; Chromatograms were obtained with absorption at 268 nm.

Reactions of BcUGTs with most of the sugar acceptors yielded single products, each of which was identified as its 7-O-glycoside on the basis of their co-chromatography with authentic samples using HPLC. However, as shown in Figure 5, when irigenin was used as acceptor substrate, BcUGT4 and BcUGT5 tended to yield double products, one of which (1a) was identified as iridin according to the mass spectrum and co-chromatography with authentic iridin, the molecular formula C_24_H_26_O_13_ was deduced from the HR-ESI-MS ion peak at *m/z* 521.1832 [M-H]^−^. The other main product (1b) was confirmed to be irigenin 3′-O glycoside using HR-ESI-MS, _1_H-NMR, _13_C-NMR, HMBC, and NEOSY. This structure has not been found previously in *I. domestica* or any other plant. Its UV (MeOH) λmax values were 268 nm, which is the typical absorption peak of isoflavones, and its IR absorptions at 3,424, 1,627, and 1,510 revealed the existence of hydroxyl and carbonyl groups (Supplementary Figure 7). The molecular formula C_24_H_26_O_13_ was deduced from the HR-ESI-MS ion peak at *m/z* 545.1275 [M+Na]^+^ (calcd for C_24_H_26_O_13_ as 522.4544) (Supplementary Figure 8). In the _1_H NMR spectrum, the most representative signal was the characteristic signal for isoflavone at δ8.43. There were three methoxyl group signals at δ3.81, δ3.76, and δ3.75. The signals at δ13.01 and δ10.93 were assigned to the hydroxy groups at the five and seven positions of isoflavone, and the signal of the end group hydrion of glucose occurred at δ4.91. The signals at δ6.96 (1H, d, 2.8) and δ6.96 (1H, d, 2.8) represented two AX coupling systems, which indicated the existence of incomplete symmetry tri-substituted groups in the B ring of the isoflavone skeleton (Supplementary Figure 9). The _13_C NMR spectrum indicated 24 carbon resonances, which contained 19 carbon resonances of the isoflavone skeleton, 3 methoxyl group signals, and 6 carbon signals of glucose (Supplementary Figure 10). In the HMBC experiment, the resonating signal at δH 4.91 correlated with δC 151.2, which indicated that the glucose was transferred to isoflavonoid 3′- hydroxyl position (Supplementary Figure 11). _1_H-NMR and _13_C-NMR data for compound 1b and key HMBC correlations were shown in Supplementary Table 5. The NEOSY spectrum showed a resonating signal correlation between two hydrogens at a distance of two chemical bonds, and the resonating signal of δH 4.91 displayed a strong correlation with δH 6.96, which implied that 1′ H of glucose was close to 2′ H in irigenin. The NEOSY result further validated the structure of 3′-O glycoside (Supplementary Figure 12).

### Kinetic Analysis of BcUGTs for Isoflavones

Although most of the BcUGTs exhibited broad substrate ranges and preference for the kaempferol aglycone, no data showed the existence of flavone O-glycosides or flavonol O-glycosides in *I. domestica*, only small amounts of flavones and flavonols have been reported, such as hispidulin, apigenin, rhamnazin, kaempferol, and quercetin (Jin et al., 2008). Consequently, we selected irigenin and tectorigenin as the substrates for kinetic parameter determinations (Table 2). The UDPG concentration was set at 2 mM, and the substrate concentration ranged from 10 to 400 μM for the assays. The reaction time was strictly controlled at 10 min. The km values of BcUGT4 and BcUGT5 for irigenin and tectorigenin were the lowest among all the active BcUGTs, and BcUGT5 exhibited the highest kcat/km ratio for irigenin, at 2.45 × 10^6^, which was approximately four times the kcat/km ratio for tectorigenin. Using BcUGT4, the kcat/km ratio for tectorigenin was approximately 4.5 times that for irigenin. Thus, although BcUGT4 shared a 94.2% sequence identity with BcUGT5, their substrate specificities were quite different. In addition, the sequence identity between BcUGT1 and BcUGT3 was 97.3%, but their catalytic specificities were totally different. BcUGT3 did not show activity against isoflavones, but BcUGT1 exhibited broad substrate ranges, although its kcat/km ratio for irigenin was just 2.8 × 10^5^. BcUGT8 shared only about 70% sequence identity with other BcUGTs, so irigenin and tectorigenin may not be it's natural substrates, and BcUGT8 could almost not glycosylate tectorigenin (Supplementary Table 4).

### Phylogenetic Analysis of BcUGTs

**Figure 6:**
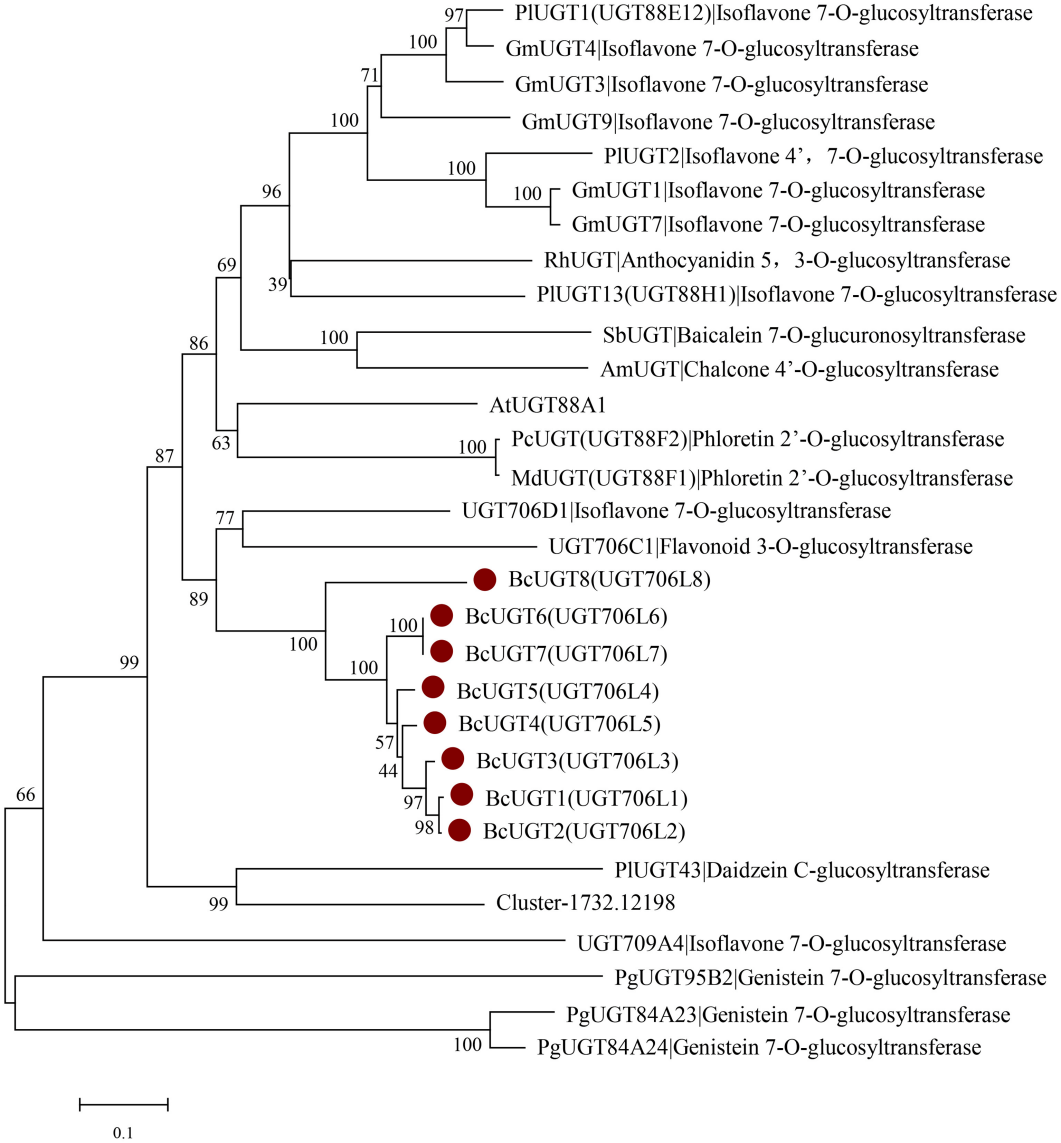
Rooted phylogenetic tree of BcUGTs, UGT88-, UGT706-, and UGT84-related (iso)flavone O-glycosyltransferase genes. *Glycine max*: GmUGT1 (A6BM07.1), GmUGT3 (NP_001353995.1), GmUGT4 (NP_001304440.2), GmUGT7 (NP_001304487.1), GmUGT9 (accession: NP_001280039.1), *Pueraria montana* var. *lobate*: PlUGT1 (A0A067YB04.1), PlUGT2 (A0A172J2D0.1), PlUGT13 (A0A067YBQ3.1), *Arabidopsis thaliana*: AtUGT88A1 (Q9LK73.1), *Scutellaria baicalensis*: SbUGT (Q76MR7.1), *Antirrhinum majus*: AmUGT (Q33DV3.1), *Rosa hybrid* cultivar: RhUGT (Q4R1I9.1), *Malus domestica*: MdUGT (B3TKC8.1), *Pyrus communis*: PcUGT (D3UAG3.1), *Punica granatum*: PgUGT95B2 (MH507175), PgUGT84A23 (KT159805.1), PgUGT84A24 (KT159807.1), *Oryza sativa* Japonica Group: UGT709A4 (BAC80066.1), UGT706D1 (BAB68093.1), UGT706C1 (BAB68090.1).

According to the phylogenetic tree, BcUGTs showed a closer relationship with the UDP-UGT706 family (Figure 6). UGT706D1 and UGT709A4 were reported to be capable of forming isoflavone 7-O glucosides *in vitro*, while UGT706C2 could use flavones to produce flavone 3-O glucosides (Ko et al., 2008), all BcUGTs showed a closer relationship with UGT706D1 and UGT706C2, and sequence identities were 40–50%. Meanwhile, BCUGTs had 40–50% amino acid sequence similarities with the UGTs of *G. max* and *P. lobata*, which have been confirmed to have activities toward (iso)flavone aglycones. Although all the BcUGTs selected for phylogenetic tree construction had similar sequence similarities with UGT88 family group and UGT706 family group, they clustered into rice UGT706 clade, perhaps owing to the genetic distance between monocots and eudicots. Genes chosen in the phylogenetic tree were mainly those of *G. max* and *P. lobata*, but they exhibited different substrate specificities and regio-selectivities. PlUGT1 and PlUGT13 showed enzyme activities for isoflavone substrates at the 7-hydroxy group, while other flavonoids were hardly accepted. PlUGT2 was later shown to be the isoflavone 4′,7-O-diglucosides' UGT. GmUGT4 was specific for isoflavones at the 7-hydroxy group, but other GmUGTs showed broad glycosyl-acceptor specificities (Li et al., 2014; Funaki et al., 2015; Wang et al., 2016). The PgUGT84A23 and PgUGT84A24 played important roles in formation of hydrolyzable tannins (HTs) in pomegranate, but they also exhibited genistein 7-O-glycosylation activity *in vitro* (Ono et al., 2016), and another PgUGT95B2 showed genistein 7-O-glycosylation activity *in vitro* as well (Wilson et al., 2019). These reports suggested BcUGTs' ability to glycosylate different flavones or isoflavones at their 7-hydroxy positions, but *in vitro* experiments revealed that their specificities and regio-selectivities for these sugar acceptors differed. A further division of the clusters among the 10 unigenes from *I. domestica* revealed that Cluster-1732.24325, Cluster-1732.17620, and BcUGT8 did not fall into the same subcluster with the other BcUGTs, and Cluster-1732.24325 and Cluster-1732.17620 had no activities toward flavones. Additionally, BcUGT8 only displayed a weak catalytic efficiency toward irigenin. BcUGT1 and BcUGT2 clustered into the same branch, and BcUGT6 and BcUGT7 displayed a close genetic relationship. Cluster-1732.12198 had a relatively higher homology to PlUGT43, which was reported to be a daidzein C-glycosyltransferase cloned from *P. lobata* (Wang et al., 2017). However, to date, there have been no isoflavone C-UGTs detected in *I. domestica*, and Cluster-1732.12198 did not transfer glucose to any flavones.

### Expression Level Analyses

**Figure 7:**
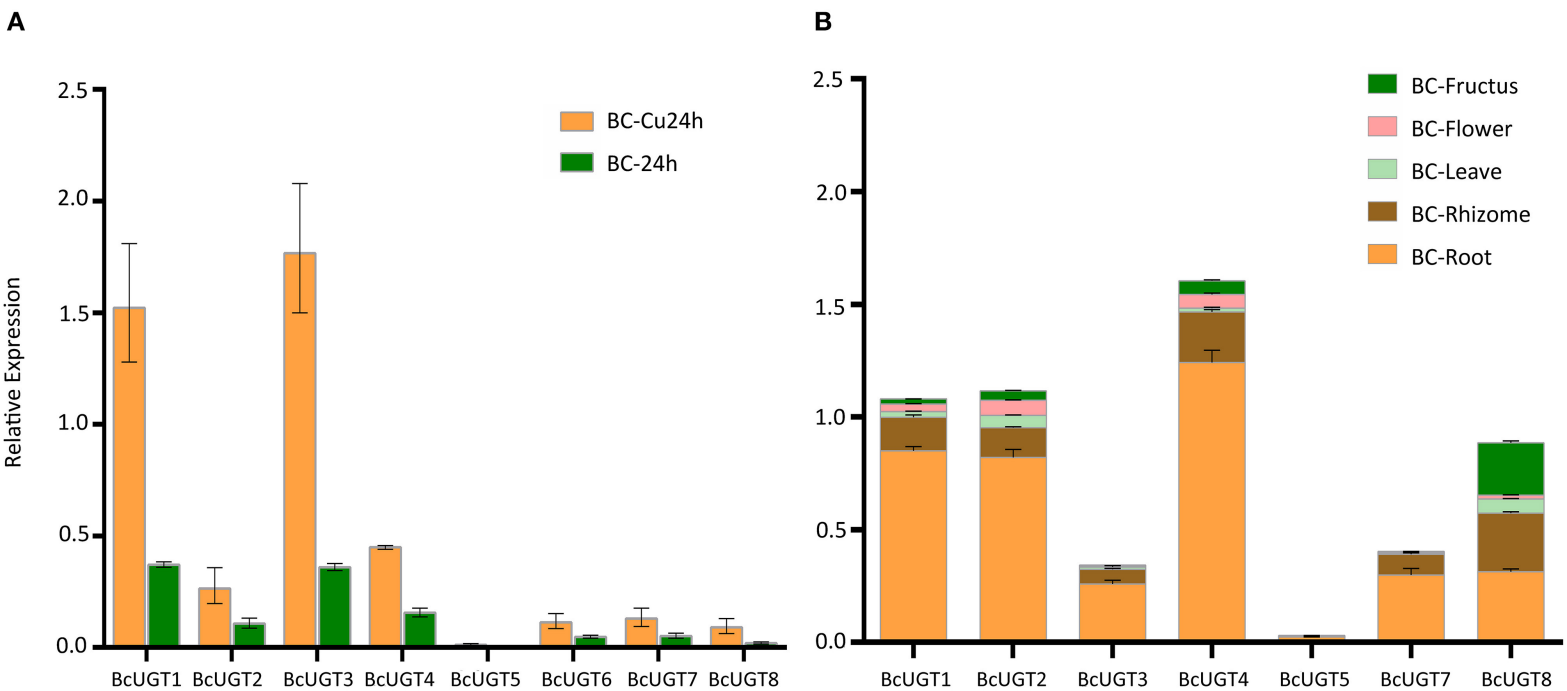
Transcriptional levels of genes coding for flavonoid-active BcUGTs. **(A)** Transcription levels of BcUGTs in the seedlings of *Iris domestica* treated with CuCl_2_ for 24 h (BC-Cu24h) and the control group (BC-24h). **(B)** Transcription levels in fructus, flower, leaf, rhizome, and root of *I. domestica* collected from the wild. Data are presented as the means ± SDs from three independent biological replicates and three technical replicates.

The transcription levels of the active BcUGTs (BcUGT1–8) in *I. domestica* seedling roots were detected using quantitative real-time RT-PCR with primers having good specificity and amplification efficiency levels (Supplementary Figure 13). After treating with CuCl_2_, the transcript abundances in seedling roots increased to different degrees, especially for BcUGT1 and BcUGT3, in addition, the expression levels of BcUGT1 and BcUGT3 were significantly higher than those of other BcUGTs (Figure 7A). The results showed good correlations with the FPKM (fragments per kilobase of exon model per million mapped reads) of each BcUGT calculated from the RNA-seq data. We found that the expression levels of all the active BcUGTs in seedlings not receiving CuCl_2_ treatments were very low, and some of them, such as BcUGT5 and BcUGT8, were almost undetectable. Thus, CuCl_2_ plays a crucial role in inducing the expression of the eight active BcUGTs. The expression levels of BcUGTs in seedlings receiving MgCl_2_, ZnCl_2_ and CaCl_2_ treatments were very low as well (Supplementary Figure 14). Moreover, ZnCl_2_ and MgCl_2_ suppressed the expression of all BcUGTs compared with the control group, CaCl_2_ showed almost no effect in inducing the expression of the eight BcUGTs. These data indicated that Cu^2+^ plays a crucial role in inducing the expression of the BcUGTs, and we could rule out the effect of the chlorine ion in the induction.

**Figure 8:**
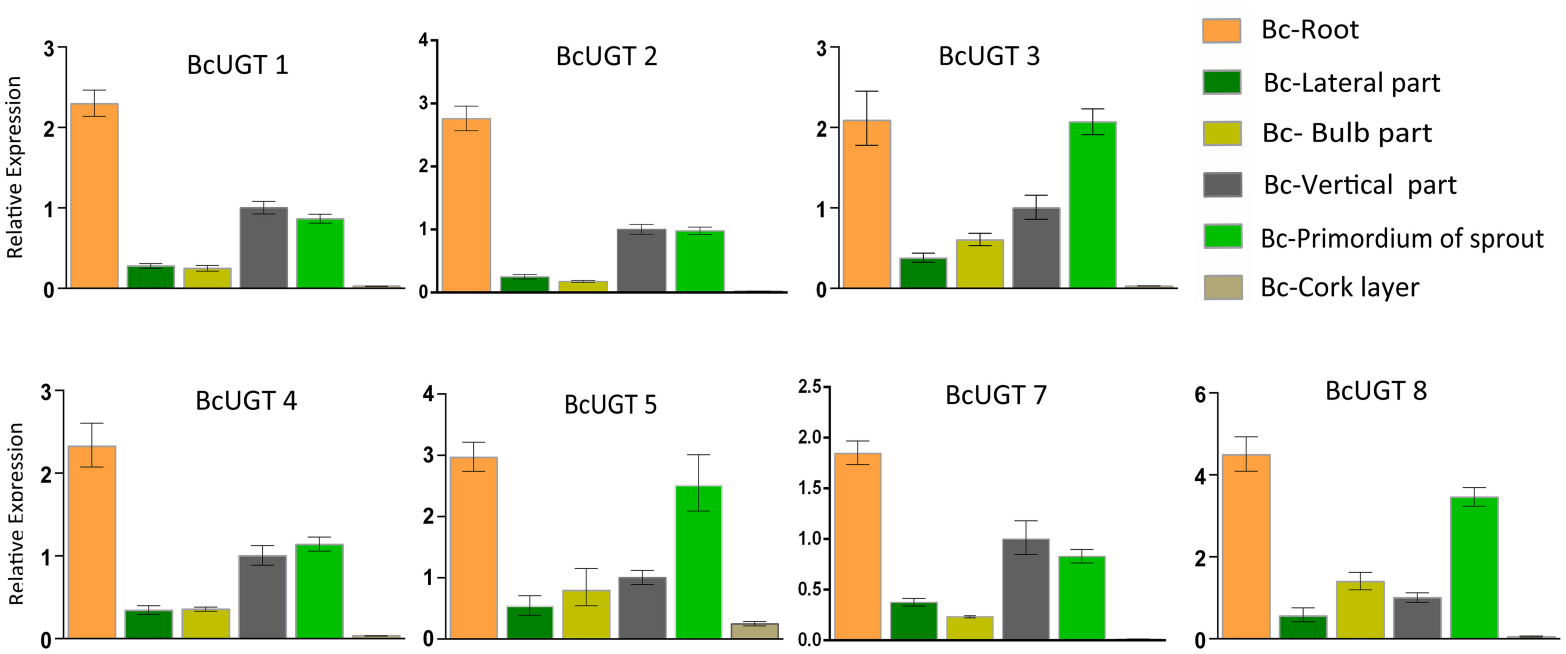
Relative transcription levels of active BcUGTs in different rhizome parts. The transcription levels of flavonoid-active BcUGTs in the lateral part, blub part, vertical part, sprout primordium, and cork layer of the rhizome. Data are presented as the means ± SDs from three independent biological replicates and three technical replicates.

The transcript abundances of all the BcUGTs in the underground parts were significantly greater than in the aerial parts, which correlated well with the isoflavone glycosides' distribution pattern (Figure 7B). The rhizome accumulated the most isoflavones, but the BcUGTs were more highly expressed in roots than rhizomes (Figure 8). To further explore the relationship of BcUGT expression levels between roots and rhizomes, we divided the triennial rhizomes into five parts, lateral, bulb, vertical, sprout primordium, and cork layer (Chen et al., 2014). The vertical part is the first and sprout primordium is the last during rhizome growth, and the lateral and bulb are biennial parts in rhizomes. Among the five rhizome parts, the sprout primordium and vertical part displayed higher BcUGT expression levels than other parts. However, it was obvious that the lateral and bulb parts had the most roots, while the other parts, especially the sprout primordium, had almost no roots. In the cork layer, previous experiments revealed that isoflavone aglycones were the most abundant and almost no isoflavone glycosides were detected (Chen et al., 2014). This may be explained by the low BcUGT expression levels.

## Discussion

To date, the biosynthesis of isoflavonoids, including isoflavone 7-O-, 4′-O-, and C-UGTs, has been mainly studied in legumes, with the only exceptions being studies from pomegranate and rice. Thus, little is known regarding the genes involved in isoflavonoid biosynthesis in other plants containing abundant isoflavones. *Iris domestica* accumulates many isoflavonoid glycosides, such as tectoridin, iridin, iristectorin A, and iristectorin B, in its rhizomes. Using copper stress, we obtained eight unigenes that were active toward (iso)flavones. A sequence alignment showed that the active BcUGTs shared a very high sequence homology (Supplementary Figure 15).

### Cu^2+^ Mechanism for Up-Regulating BcUGT Expression

Plants are continuously exposed to abiotic stresses during their growth in the wild, including various heavy metal stresses (Piscopo et al., 2016). Consequently, they have evolved a series of skills to deal with the rapid changes in the environment. Elements such as Fe, Cu, Zn, Mo, and Ni are considered to be essential heavy metals (HMs) (Hall, 2002; Arif et al., 2016), but excessive HMs may be toxic for plants, resulting in large-scale molecular, biochemical and physiological responses (Hayat et al., 2012; Hussain et al., 2013; Clemens and Ma, 2016). The perception of stress stimuli is the initial plant response when exposed to toxic HMs. Then, the amplified signal is transduced and transmitted into cells to bring about cascade reactions of molecular, biochemical and physiological activities (Wani et al., 2018). Through transcription analyses of *I. domestica* roots, many P450 genes related to the biosynthesis and catabolism of methyl jasmonate and allene oxide (Maksymiec et al., 2005), including CYP94 (cluster1732.31596 and cluster1732.31816) and CYP74 (cluster 1732.20141), were found to be up-regulated after CuCl_2_ treatments (Utsunomiya et al., 2000; Koo et al., 2011).

At the biochemical level, plants need more secondary metabolites to cope with excess HMs, resulting in the high expression levels of many unigenes involved in the biosynthesis of secondary metabolites. CuCl_2_ was first applied to induce isoflavonoids in *P. lobata* stems, and the daidzein and genistein contents in the CuCl_2_-treated stem increased 5–10-fold compared with the control group (Hakamatsuka et al., 1991). The cotyledons of *Lupinus albus* have been treated with a fungal elictor and CuCl_2_, which induced the isoflavone 2′-hydroxygenistein. When challenged with CuCl_2_, isoflavone glycosidic conjugates are largely degraded to isoflavone aglycones, which indicates the important roles of isoflavonoid glycosides in plants coping with Cu^2+^ toxicity (Wojtaszek and Stobiecki, 1997). Additionally, the dynamic change in isoflavonoid glycosides contents in *I. domestica* calli suggested that CuCl_2_ promotes the biosynthesis of isoflavone glycosides during the first stage of CuCl_2_ stress. Afterward, when the Cu^2+^ concentration in cells reaches a high level, the isoflavone glycoside is hydrolyzed to its aglycone. Thus, we speculated that isoflavone glycosides play very important roles in plant defenses against Cu^2+^ stress. Through the differential expression analysis, several up-regulated unigenes annotated as (iso)flavone 7-O-UGTs, which belonged to the UGT706 family, were cloned. Then, these unigenes were expressed in a recombinant *E. coli* expression system, and their functions were verified using *in vitro* catalytic experiments. These results implied that the high CuCl_2_ concentration enhanced the expression of (iso)flavone O-UGTs in *I. domestica*.

### Substrate Preference and Regio-Specificity of BcUGTs

Many flavone UGTs show more regio-specificity than substrate specificity (Kim et al., 2006; Li et al., 2014; Funaki et al., 2015; Wang et al., 2016). In soybean, GmUGT1 and GmUGT7 efficiently catalyze regio-specific glucosyl transfers to isoflavones at their 7-hydroxy positions, and they accept naringenin, apigenin, quercetin, and kaempferol as substrates (Funaki et al., 2015). In an important crop, *Camellia sinensis*, CsUGT73A20 was identified as a flavonoid UGT. CsUGT73A20 transfers UDP-glucose to produce 3-O- and 7- O-glycosides, depending mainly on the reaction's *in vitro* pH value. Kaempferol is the most preferred substrate, but naringenin and apigenin are also accepted (Zhao et al., 2017). Recently, three new (iso)flavonoid glycosyltransferases (PlUGT4, −15 and −57) were identified in *P. lobate*. PlUGT15 and PlUGT57 exhibit much higher isoflavone specificity than other 7-O-UGTs found in *P. lobate* (Wang X. et al., 2019).

**Figure 9:**
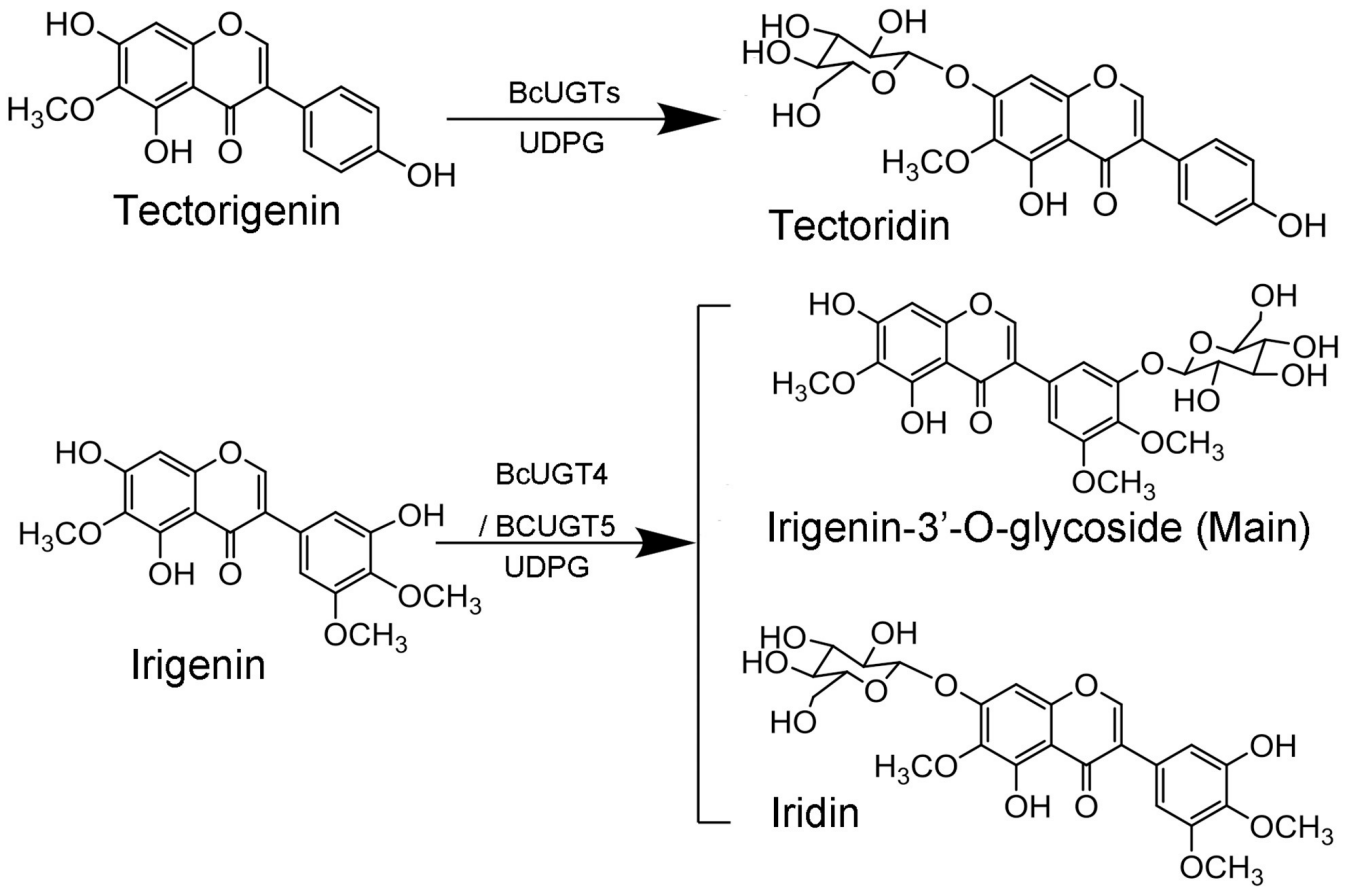
Isoflavone O-glucosyltransferase-catalyzed reaction.

Kaempferol is the most preferred acceptor among the 12 substrates tested for all the BcUGTs. The major product yielded by BcUGTs was (iso)flavone 7-O-glycoside, which indicates that the BcUGTs preferred to transfer the sugar moiety to the hydroxy of ring A, but no (iso)flavone 4′-O-glycosides or 4′,7-O-di-glycosides were obtained. Thus, most of the BcUGTs had no activity toward the hydroxy of ring B. Tectorigenin is enriched in the rhizomes and roots of *I. domestica*, and all the BcUGTs, except BcUGT3 and BcUGT8, converted tectorigenin to tectoridin, which exists in large amounts in *I. domestica*. Irigenin is another main isoflavone aglycone in the rhizomes and roots of *I. domestica*, but we unexpectedly found that the main product of BcUGT4 and BcUGT5 using irigenin as the substrate was not iridin (Figure 9). We identified its structure as irigenin 3′-O-glycoside. This is a new compound that has never been identified in *I. domestica* or other plants. However, other BcUGTs hardly glycosylated irigenin at the 3′-OH position. BcUGT4 yielded two different products using luteolin as the substrate, but the main product was luteolin-7-O-glycoside. Thus, we speculated that the binding conformation of the substrate determines the glycosylation position for BcUGT4 and BcUGT5.

Interestingly, substrates lacking a 5-hydroxy were hardly glycosylated by BcUGTs. Recently, a promiscuous flavonoid-3-O-UGT (sb3GT1) was identified in *Scutellaria baicalensis*, and it accepts many sugar donors to glycosylate 17 flavonols. Through homologous molecular modeling and docking, 18 residues within a 5-Å distance of the binding pocket were selected as candidates, and residue G15 was identified as the critical residue in the sugar donor promiscuity of Sb3GT1 by comparisons with the docking results of *Vitis vinifera* GT1 (Wang Z. L. et al., 2019). In this study, the docking results of BcUGT4 with liquiritigenin and naringenin indicated that the residues GLN-394 and TYR-391 could form hydrogen bond with 5-hydroxy, which may stabilize the substrate in the binding pocket, further study could confirm the role of the 5-hydroxy and the coordinating amino acids in substrate binding.

### Predicting the Function of BcUGTs

The different expression levels of BcUGTs in the transcriptome of *I. domestica* seedlings indicate that BcUGTs may play important roles in responses to abiotic stresses. In plants, glycosylation is a key modification that allows the formation of a myriad of secondary metabolites, which play many important roles in plant responses to challenges faced during growth and development (Bowles et al., 2005; Gachon et al., 2005; Campos et al., 2019). PlUGT4, −15 and −57 are up-regulated in *P. lobata* roots after methyl jasmonate treatments (Wang X. et al., 2019), and PlUGT1 may be related to the defense response against the MeJA treatment. In this paper, all the BcUGTs were up-regulated after CuCl_2_ stress, especially BcUGT1 and BcUGT2, which have highly homologous nucleotide sequences and broad substrate ranges. The unigenes encoding the core metabolic pathway usually form small families consisting of unique genes. For example, isoflavone synthases belong to the CYP93C family, which only consists of a few members in leguminous plants (Jung et al., 2000). This is in sharp contrast to the multiple gene families of UGTs, which tend to contain hundreds of members. In addition, the broad substrate ranges may ensure that plants respond rapidly to both abiotic and biotic stresses in the environment.

For wild *I. domestica*, we found that the transcription levels of most BcUGTs in the vertical parts (triennial rhizome) and sprout primordium were greater than in the lateral and bulb parts (biennial rhizome). Additionally, we found that lateral and bulb parts had more roots than the other parts; consequently, we speculated that this is the reason for the differences in BcUGT expression among these rhizome parts. Roots form the main organ that delivers water and nutrients from the soil to the aerial plant parts; therefore, we hypothesized that many isoflavone glycosides synthetized in roots by BcUGTs are delivered to the lateral and bulb parts, while the sprout primordium and vertical part have no isoflavone glycoside source. Consequently, they have to increase the BcUGT expression levels to defend against the stress. Recently, isoflavone membrane transformation in *Trifolium pratense* was shown to be related to the presence of ABC proteins and vesicular transport (Kubes et al., 2019). By analyzing global transcriptomes of six organs from *I. domestica*, many ABC transporter family unigenes were obtained specifically from the roots.

## Data Availability Statement

The sequencing project has been deposited at the SRA database under the accession number PRJNA596865. The SRA accession numbers are SRR10743030 and SRR107430. The accession numbers for the eight BcUGTs are as follows: BcUGT1 (MN894539), BcUGT2 (MN894540), BcUGT3 (MN894541), BcUGT4 (MN894542), BcUGT5 (MN894543), BcUGT6 (MN894544), BcUGT7 (MN894545), and BcUGT8 (MN894546).

## Author Contributions

XZ, YaZ, GX, and MQ designed research. XZ, JY, and ZY performed research and all authors analyzed the data. XZ, YaZ, YuZ, and MQ wrote the paper. All authors read and approved the final manuscript.

## Conflict of Interest

The authors declare that the research was conducted in the absence of any commercial or financial relationships that could be construed as a potential conflict of interest.

## Abbreviations

cDNA: complementary DNA
CuCl_2_: copper chloride
UDPG: uridine diphosphate glucose
UGT: UDP-sugar glycosyltransferase.
