# Supplementary material for: Iris domestica (iso)flavone 7- and 3′-O-Glycosyltransferases Can Be Induced by CuCl2
Source: Front Plant Sci. 2021 Feb 9;12:632557. doi: 10.3389/fpls.2021.632557 (PMC7900552; doi:10.3389/fpls.2021.632557)
Supplement: Supplementary file 1 [file Data_Sheet_1.PDF]

Supplementary Figures

Figure S1 Quality assessment of total RNA used for De Novo Sequencing 2
Figure S2 Classification of annotated unigenes by gene function 3
Figure S3 KEGG classification of annotated unigenes 4
Figure S4 SDS-PAGE analysis of the recombinant BcUGTs 5
Figure S5 Structures of flavonoids used in this study 6
Figure S6 HPLC analyses of reactions of BcUGTs with various substrates 7
Figure S7 UV and IR absorption of catalytic product of BcUGT4 for irigenin 9
Figure S8 Mass spectrum of catalytic product.. 10
Figure S9 $_{1}$H NMR spectrum of 1b in DMSO-*d6* (500 MHz).................................... 11
Figure S10 $_{13}$C NMR spectrum of 1b in DMSO-*d6* (125 MHz)................................. 11
Figure S11 HMBC spectrum of 1b in DMSO-*d6* (500 MHz). 12
Figure S12 NOESY spectrum of 1b in DMSO-*d6* (500 MHz)................................... 12
Figure S13 Melt curves of quantitative real-time PCR products in this study 13
Figure S14 Transcription levels of eight BcUGTs in the roots of *Iris domestica* seedlings treated with $ZnCl_2$, $CaCl_2$, $MgCl_2$ and $CuCl_2$ 15
Figure S15 Alignment amino acid sequences of BcUGTs 16

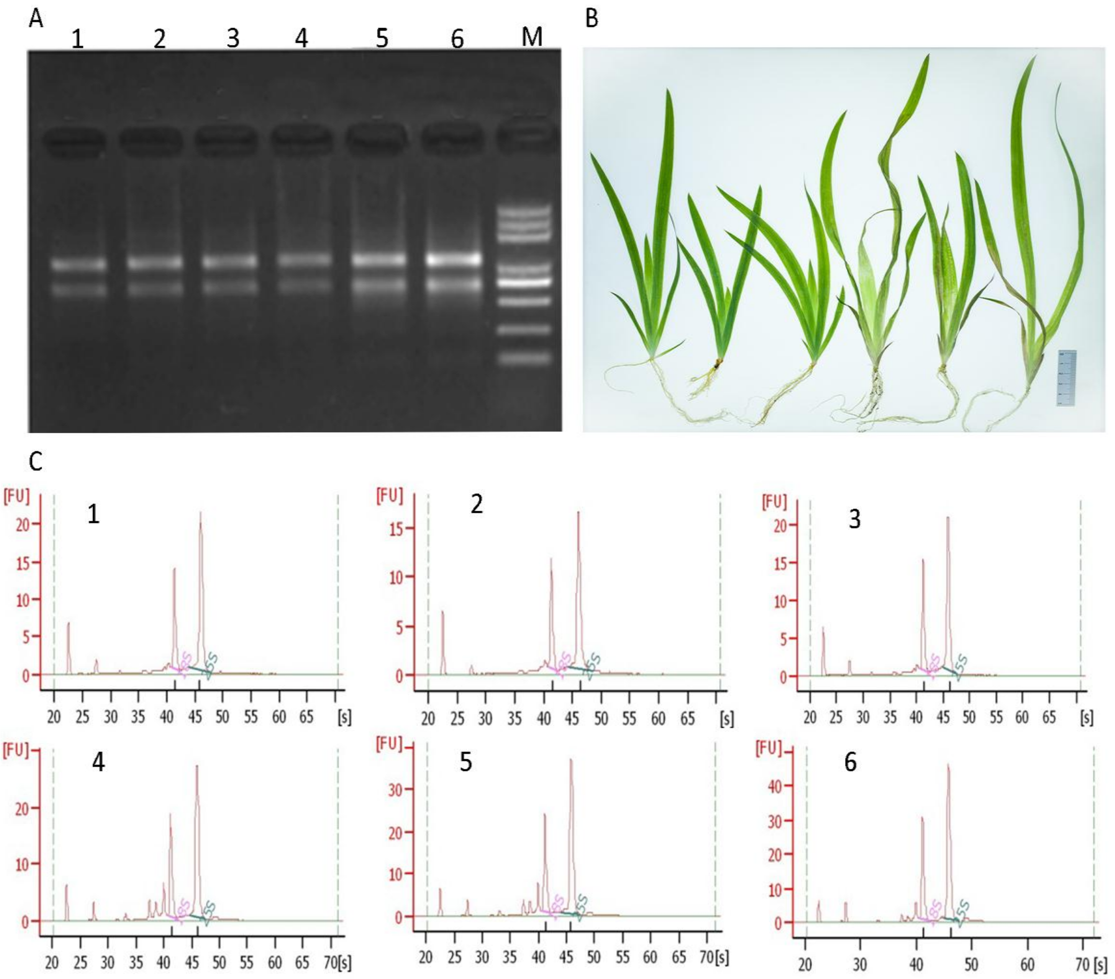

D

No.	Samples	Total(ug)	OD260/280	OD260/230	RIN
1	BC24-1	2.13	1.92	0.33	9.1
2	BC24-2	2.24	1.82	0.17	8.7
3	BC24-3	2.17	2.43	0.94	9.2
4	Cu24-1	2.18	1.88	0.81	9.0
5	Cu24-2	2.24	1.84	0.46	9.2
6	Cu24-3	2.11	2.20	0.30	9.3

Figure S1 Quality assessment of total RNA used for *de novo* sequencing.
(A) Agarose gel electrophoresis of total RNA. **(B)** Samples used for sequencing (left three: control group; right three: $CuCl_2$ treatment group). **(C)** Total RNA determined by HPLC. **(D)** Summary sheet of extracted RNAs. 1,2,3: samples of control; 4,5,6: samples of $CuCl_2$ treatment.

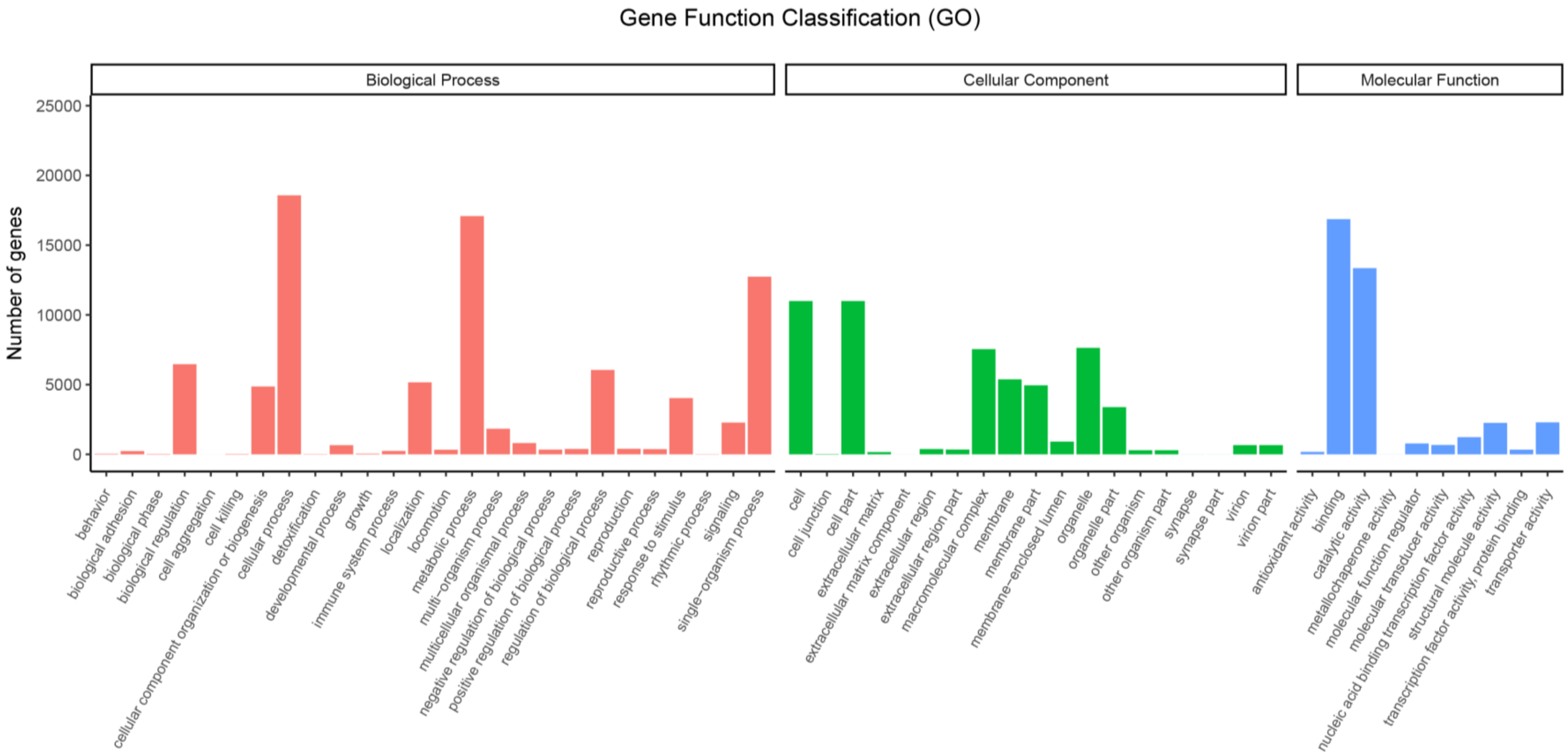

Figure S2 Classification of annotated unigenes by gene function.

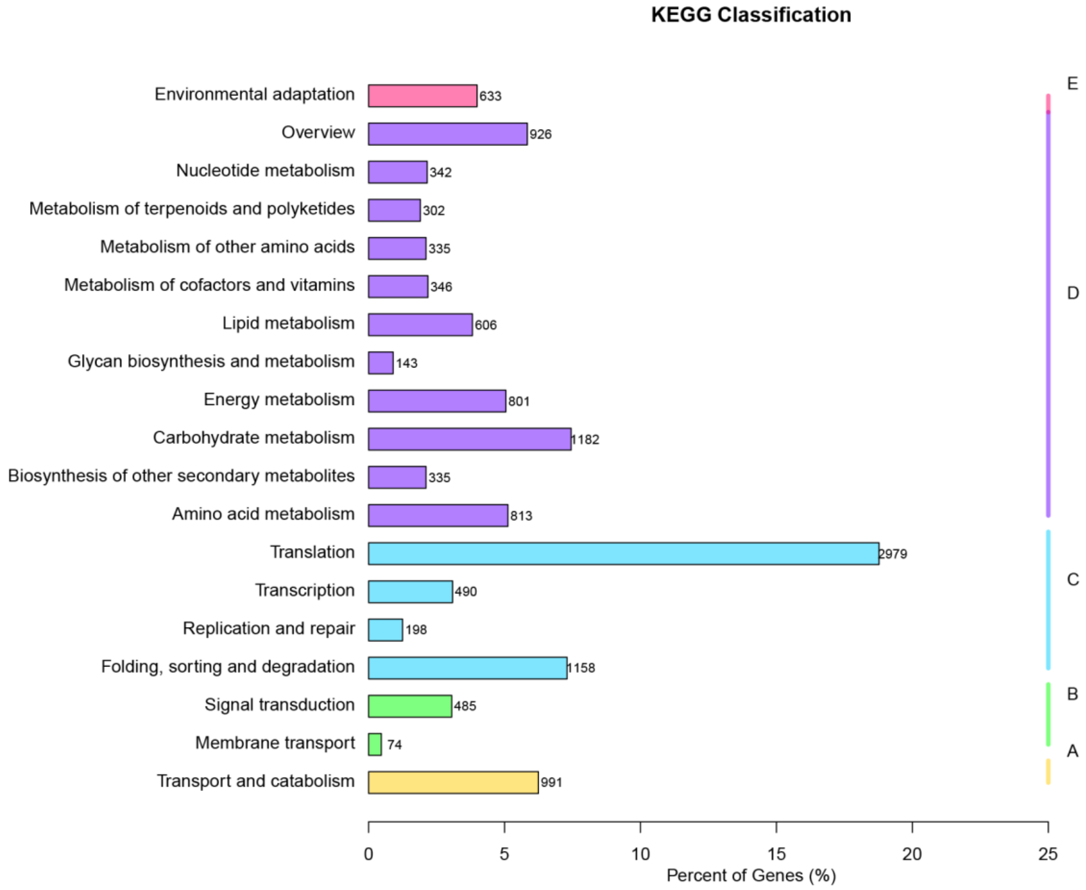

Figure S3 KEGG classification of annotated unigenes.

(A) Cellular Processes; **(B)** Environmental Information Processing; **(C)** Genetic Information Processing; **(D)** Metabolism; **(E)** Organismal Systems.

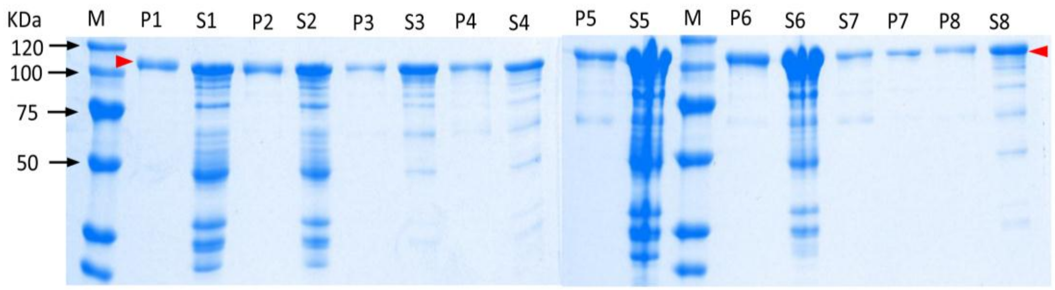

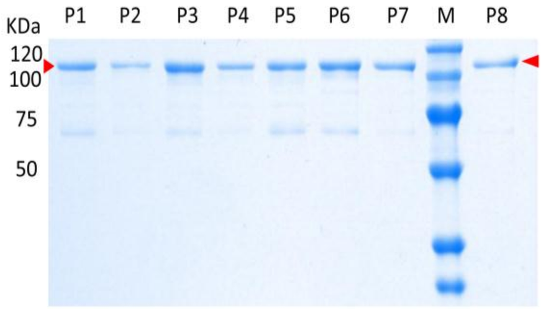

Figure S4 SDS-PAGE analysis of the recombinant BcUGTs.

Crude extract of recombinant *E,coil* cells and eluted fractions by Ni-NTA column were subjected to SDS-PAGE. S1 to S8, crude extract of the recombinant *E. coli* cells of BcUGT1 to BcUGT8, respectively; P1 to P8, fractions of elution with 5 ml buffer containing 250 mM imidazole of BcUGT1 to BcUGT8. Protein bands of BcUGTs are indicated by red arrowheads. BcUGT 1 to 8 are active (iso)flavone glycotransferases cloned from *I. domestica*.

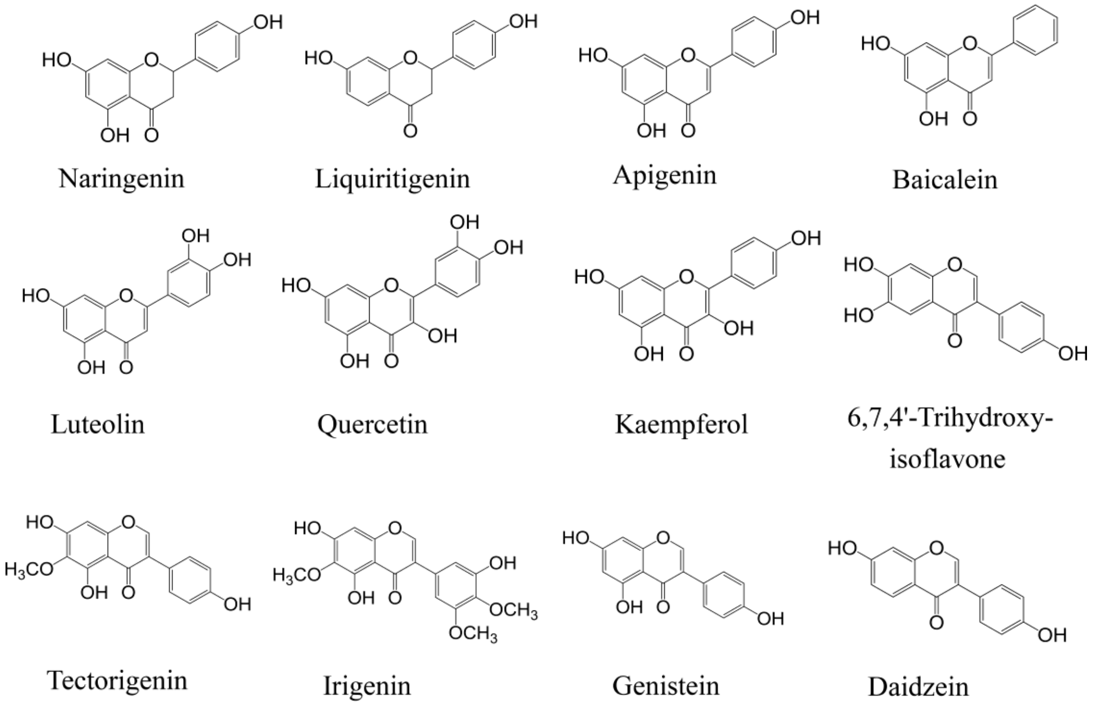

Figure S5 Structures of flavonoids used in this study.

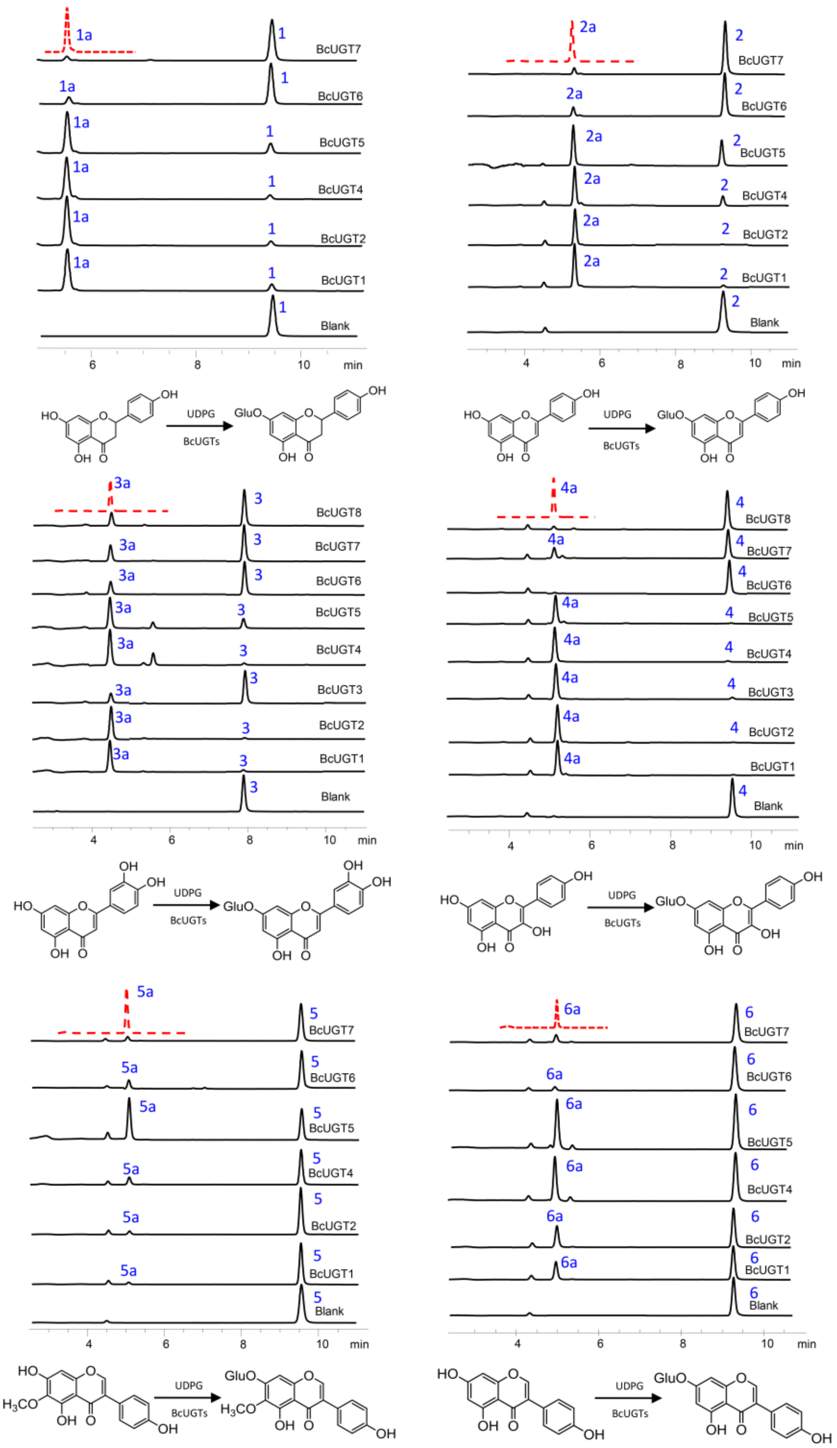
1a
1
BcUGT7
BcUGT6
BcUGT5
BcUGT4
BcUGT2
BcUGT1
Blank
6
8
10
min
2a
2
4
HO
OH
UDPG
BcUGTs
GluO
3a
3
BcUGT8
BcUGT3
4a
5a
5
H3CO
6a
6

Figure S6 HPLC analyses of reactions of BcUGTs with various substrates.

(A) A reaction mixture of naringenin and UDP-glucose with BcUGTs. 1, Naringenin; 1a, Naringenin-7-O-glycoside; Red dashed line, Authentic naringenin-7-O-glycoside; Chromatograms were obtained at 280 nm;
(B) A reaction mixture of apigenin and UDP-glucose with BcUGTs. 2, Apigenin; 2a, Apigenin-7-O-glycoside; Red dashed line, Authentic apigenin-7-O-glycoside; Chromatograms were obtained at 340 nm;
(C) A reaction mixture of luteolin and UDP-glucose with BcUGTs. 3, Luteolin; 3a, Luteolin-7-O-glycoside; Red dashed line, Authentic luteolin-7-O-glycoside; Chromatograms were obtained at 340 nm;
(D) A reaction mixture of kaempferol and UDP-glucose with BcUGTs. 4, Kaempferol; 4a, Kaempferol-7-O-glycoside; Red dashed line, Authentic kaempferol-7-O-glycoside; Chromatograms were obtained at 360 nm;
(E) A reaction mixture of tectorigenin and UDP-glucose with BcUGTs. 5, Tectorigenin; 5a, Tectorigenin-7-O-glycoside; Red dashed line, Authentic tectorigenin-7-O-glycoside; Chromatograms were obtained at 268 nm;
(F) A reaction mixture of genistein and UDP-glucose with BcUGTs. 6, Genistein; 6a, Genistein-7-O-glycoside; Red dashed line, Authentic genistein-7-O-glycoside; Chromatograms were obtained at 268nm.

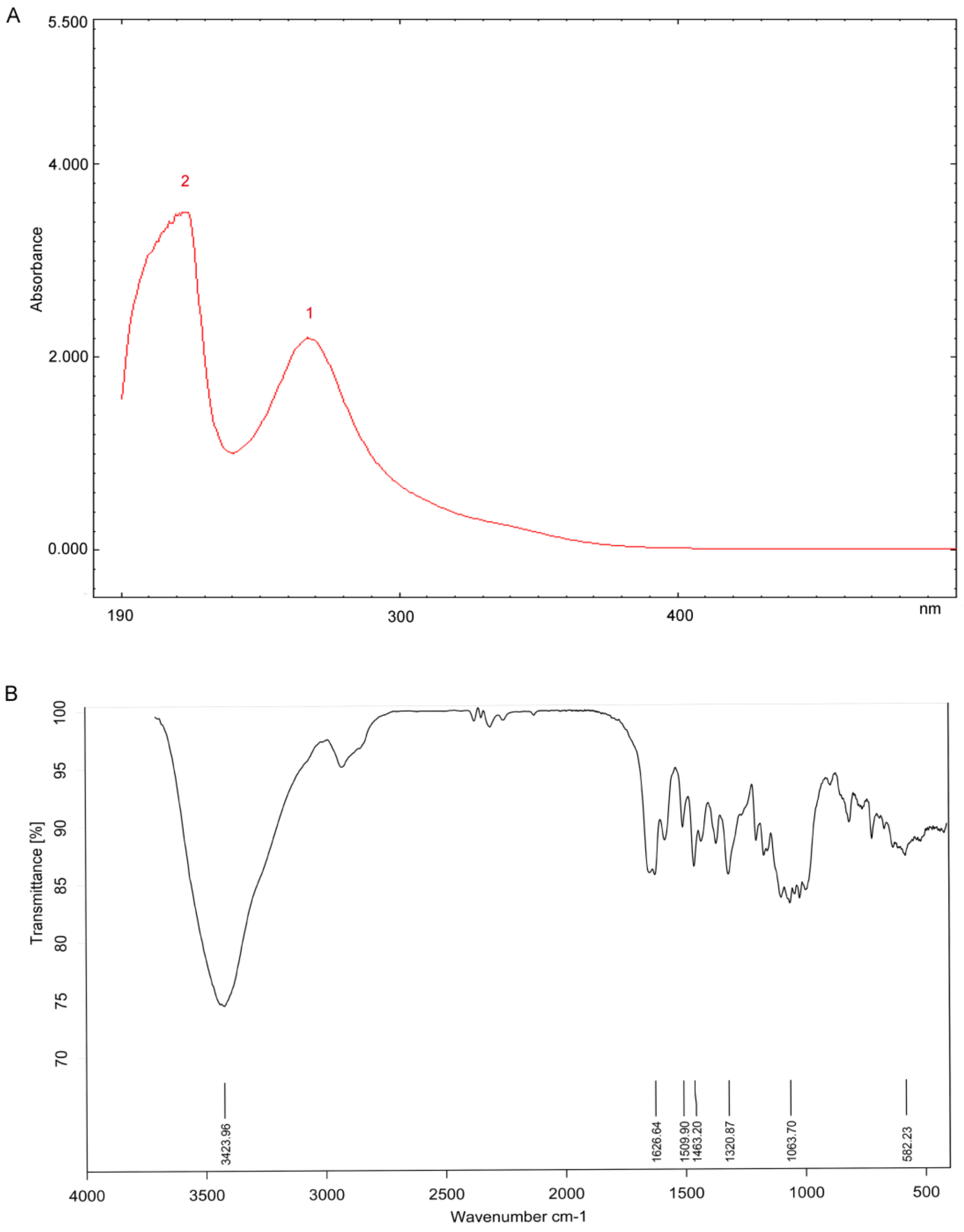

Figure S7 UV and IR absorption of catalytic product of BcUGT4 for irigenin.
(A) Full wavelength scanning of irigenin-3'-O-glycoside (methol) from 190nm to 500 nm; Peak 1, 268 nm; Peak2, 217 nm. (B) IR absorption of irigenin-3'-O-glycoside (KBr) with fourier transform infrared spectra (FT-IR).

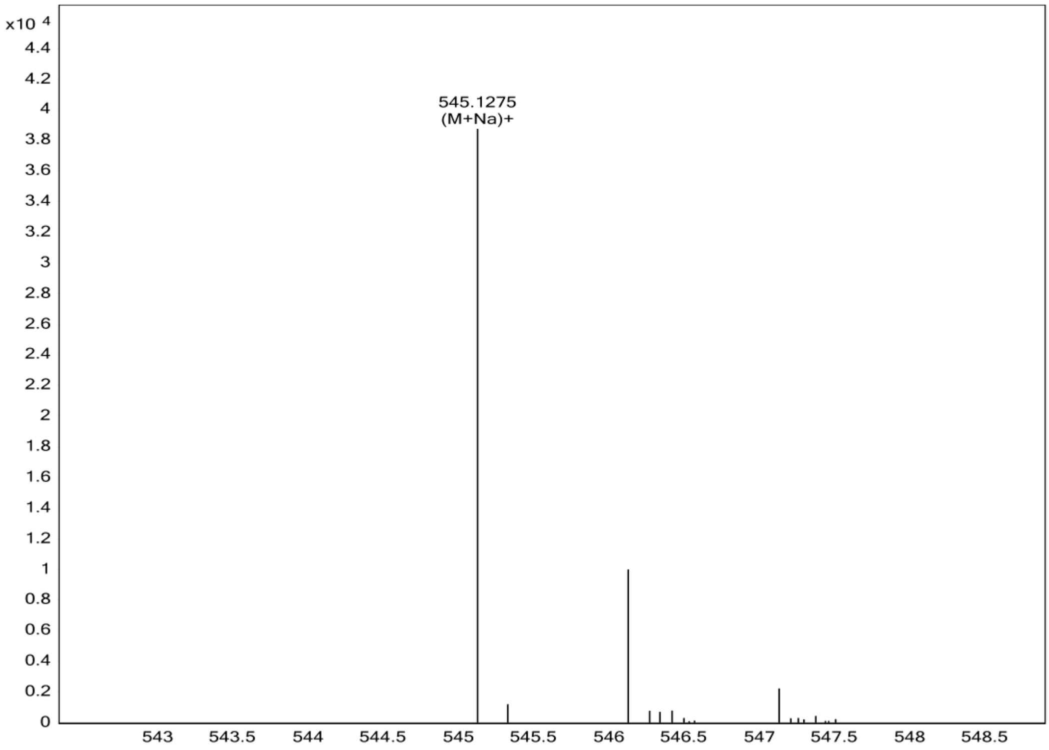

Figure S8 Mass spectrum of catalytic product (Irigenin-3'-O-glycoside, $C_{24}H_{26}O_{13}$).

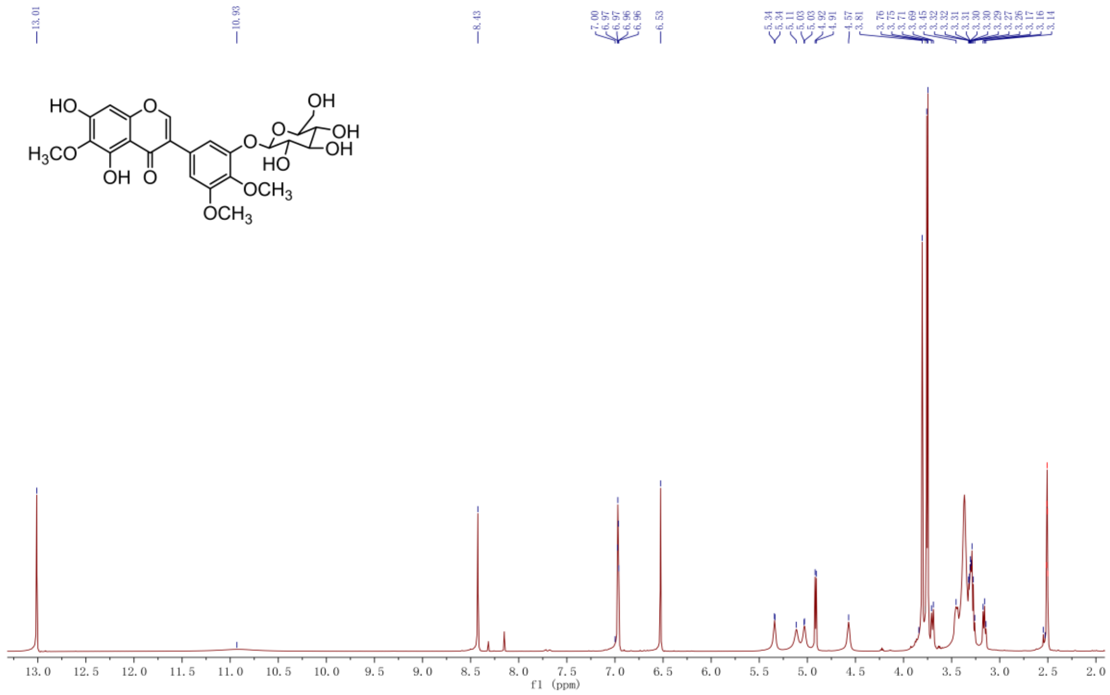

Figure S9 $_1$H NMR spectrum of 1b in DMSO-*d6* (500 MHz).

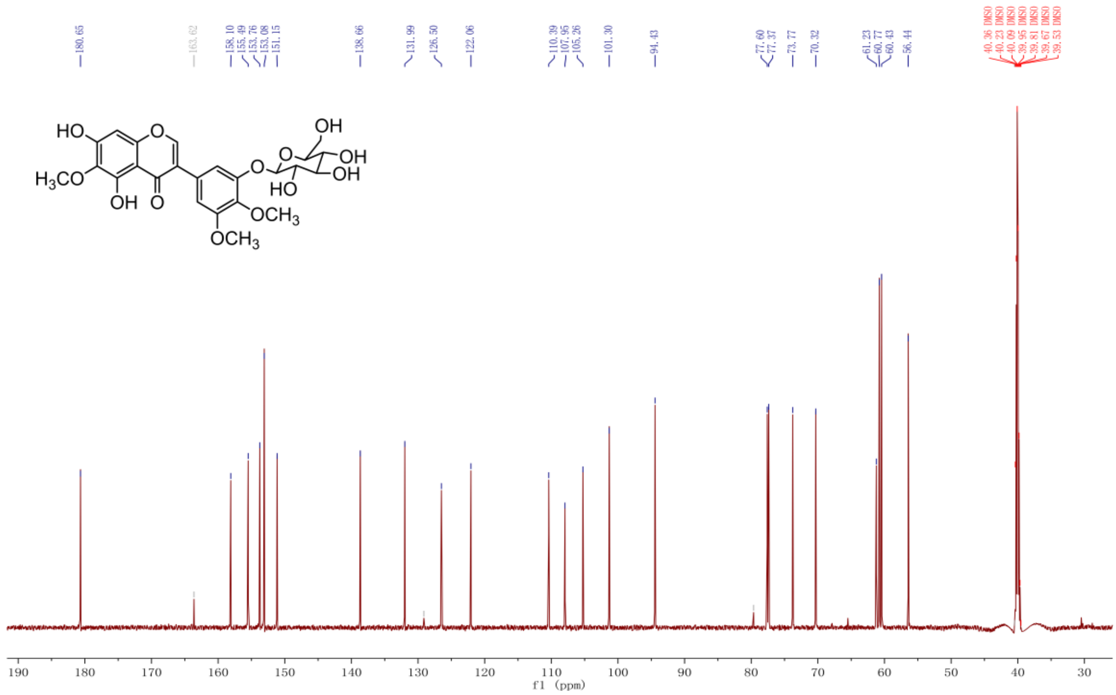

Figure S10 $_{13}$C NMR spectrum of 1b in DMSO-*d6* (125 MHz).

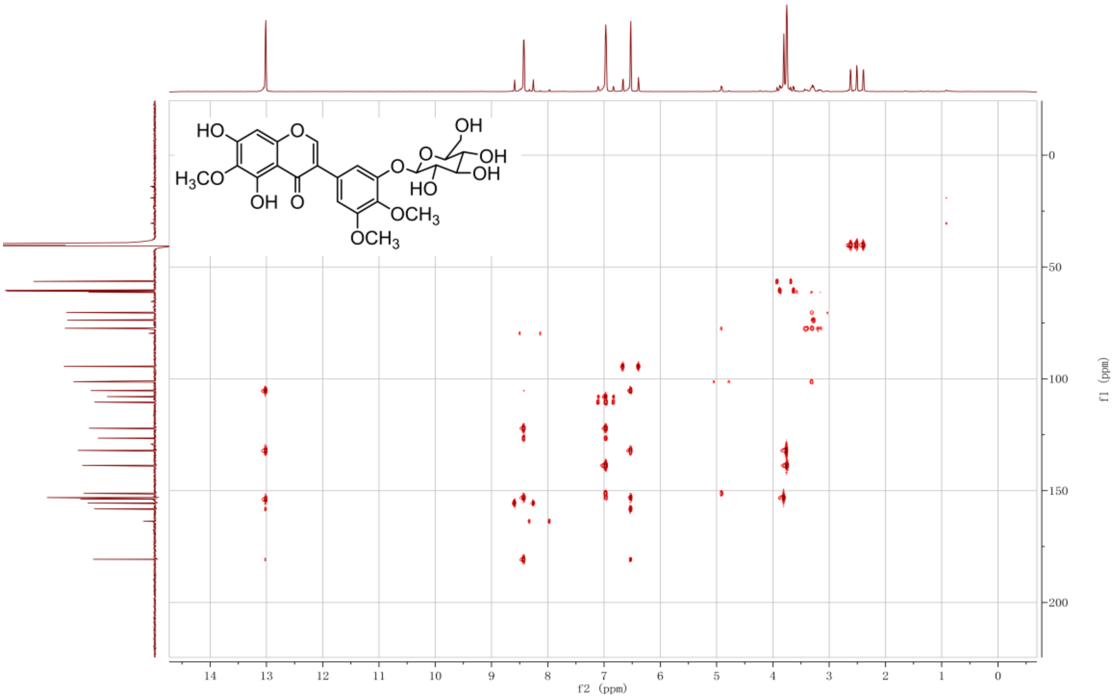

Figure S11 HMBC spectrum of 1b in DMSO-*d6* (500 MHz).

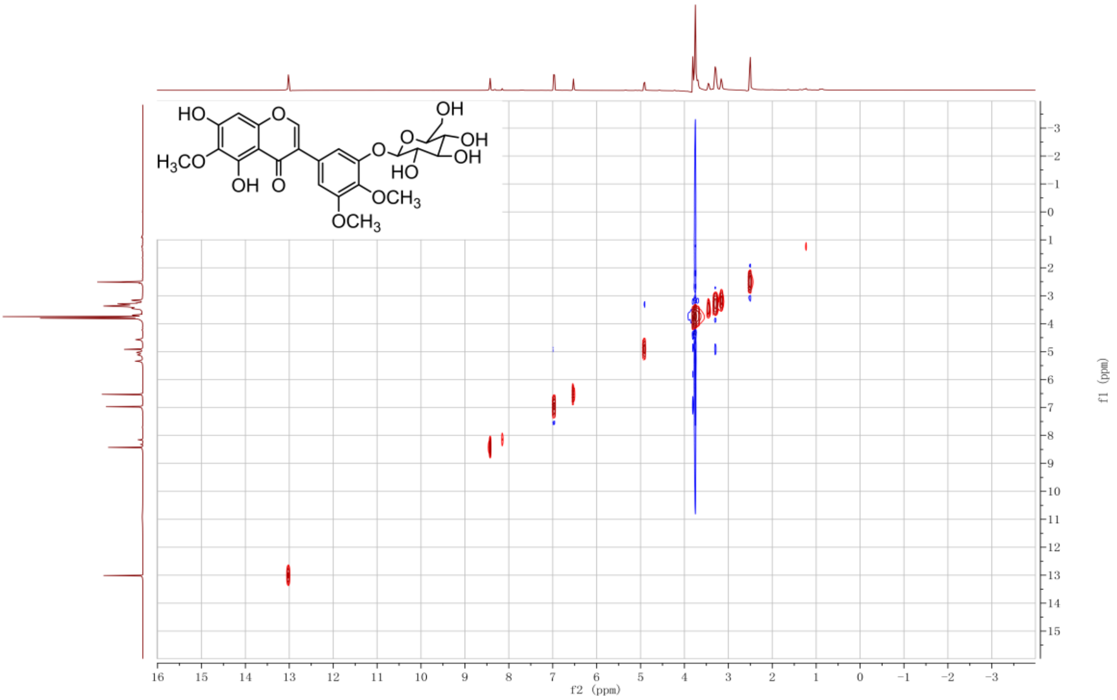

Figure S12 NOESY spectrum of 1b in DMSO-*d6* (500 MHz).

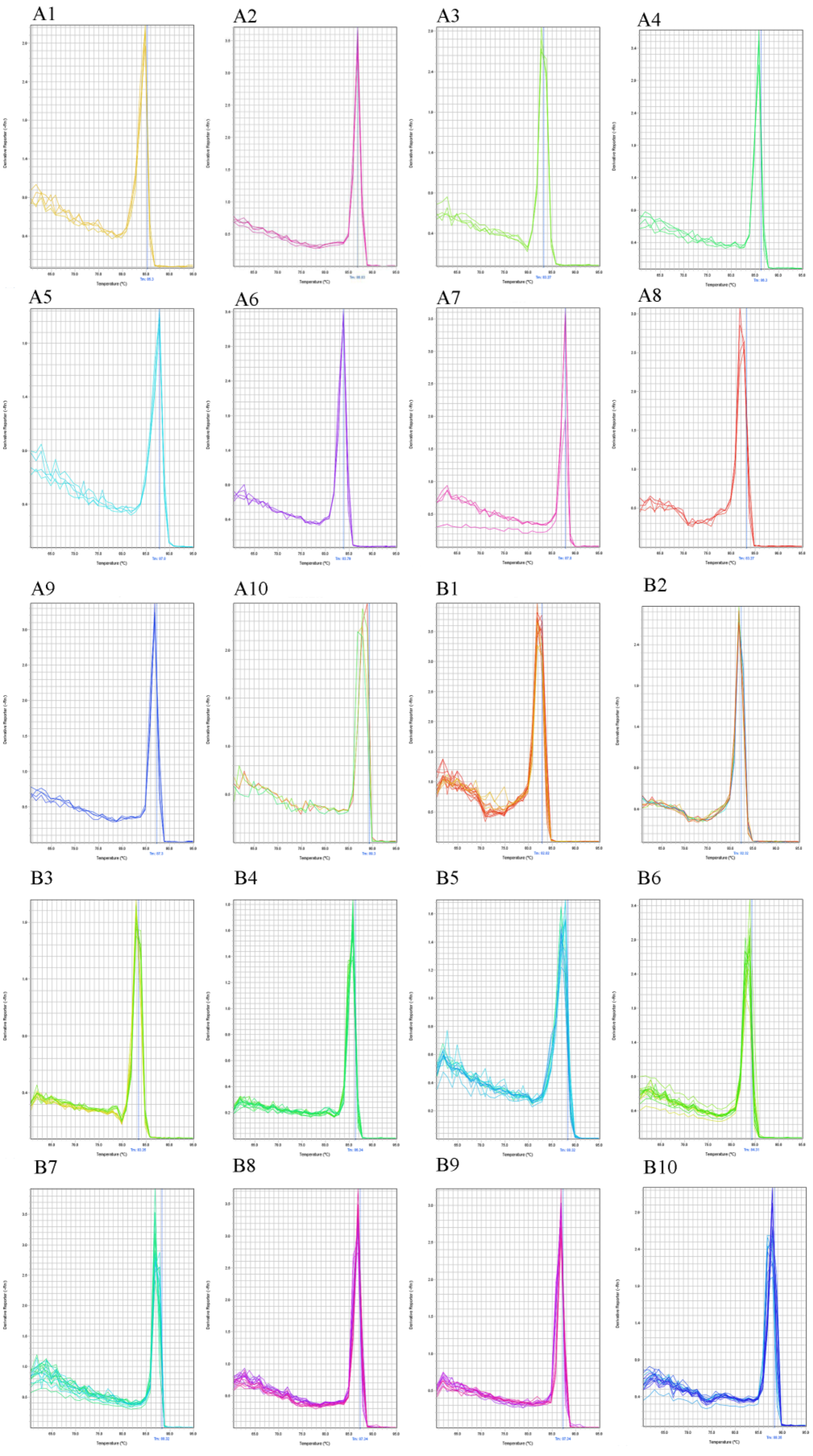
A1
A2
A3
A4
A5
A6
A7
A8
A9
A10
B1
B2
B3
B4
B5
B6
B7
B8
B9
B10

Figure S13 Melt curves of quantitative real-time PCR products in this study.

A1, GADPH for BC24 and Cu24; **A2**, Actin for BC24 and Cu24; **A3**, BcUGT1 for BC24 and Cu24; **A4**, BcUGT2 for BC24 and Cu24; **A5**,BcUGT3 for BC24 and Cu24; **A6**, BcUGT4 for BC24 and Cu24; **A7**, BcUGT5 for BC24 and Cu24; **A8**, BcUGT6 for BC24 and Cu24; A9, BcUGT7 for BC24 and Cu24; A10, BcUGT8 for BC24 and Cu24; **B1**, GADPH for different organs from *I. domestica* ; **B2**, Actin for different organs; **B3**, BcUGT1 for different organs ; **B4**, BcUGT2 for different organs; **B5**, BcUG3 for different organs; **B6**, BcUGT4 for different organs; **B7**, BcUGT5 for different organs; **B8**, BcUGT6 for different organs; **B9**, BcUGT7 for different organs;B10, BcUGT8 for different organs. Every unigene was detected in three independent biological replicates and three technical replicates.

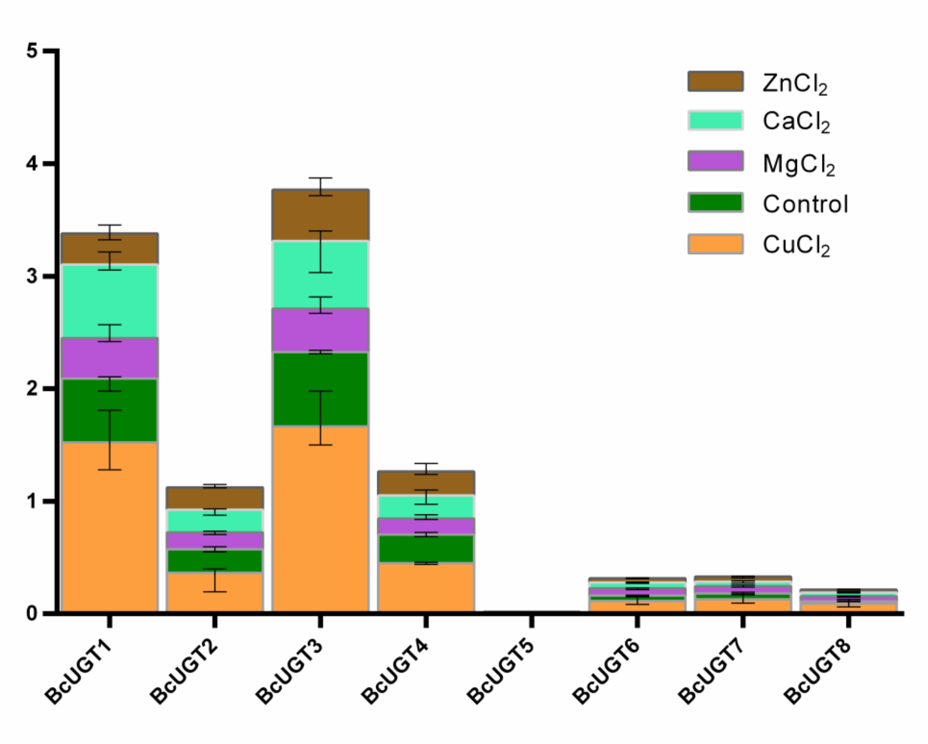

Figure S14 Transcription levels of eight BcUGTs in the roots of *Iris domestica* seedlings treated with $ZnCl_2$, $CaCl_2$, $MgCl_2$ and $CuCl_2$. Data are presented as the means ± SDs from three independent biological replicates and three technical replicates.

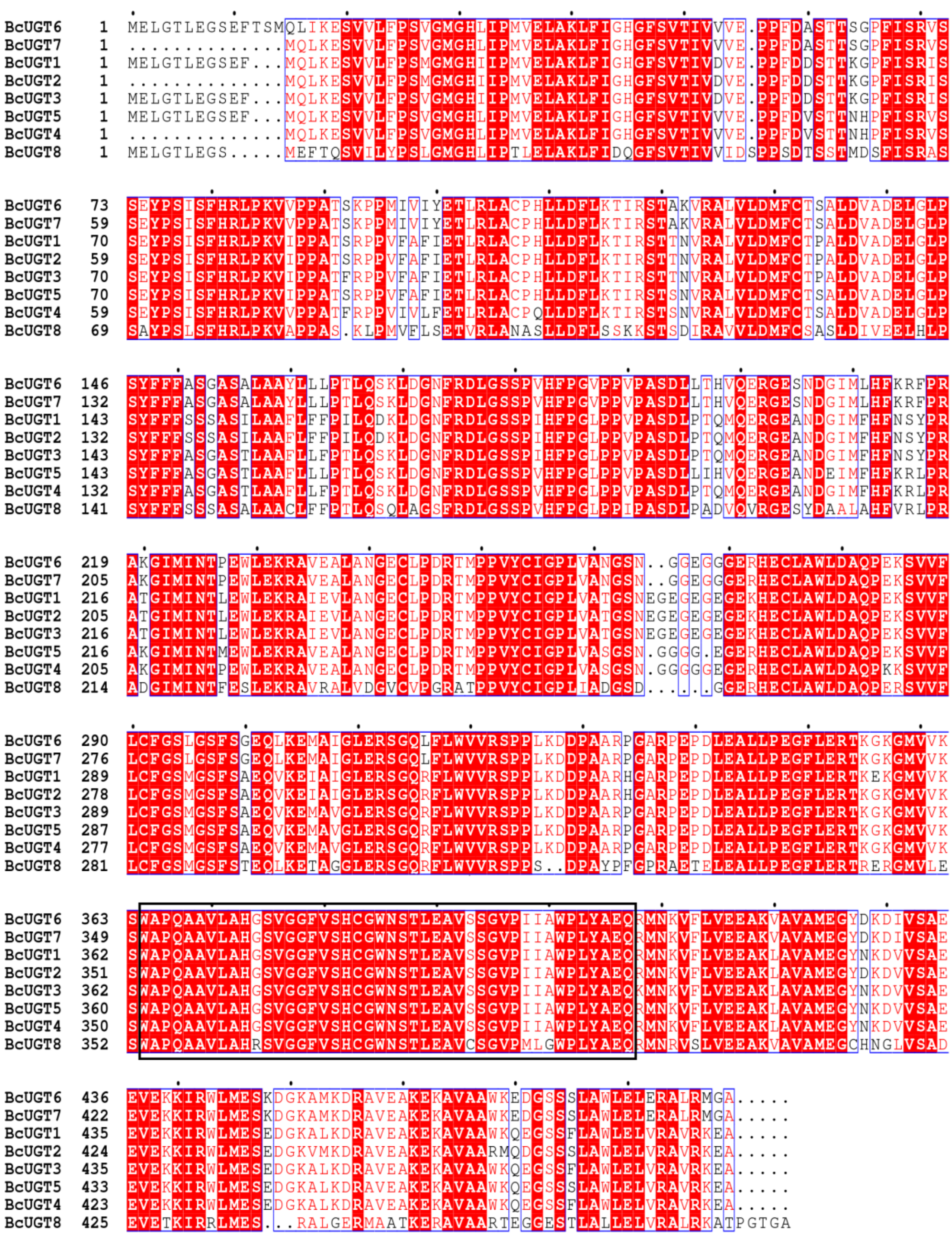

Figure S15 Amino acid sequences alignment of BcUGTs.

100% homology amino acid residues are marked with red shading, residues with >70% homology are in red front. The PSPG box of BcUGTs were marked in black box.
