# Supplementary material for: Iris domestica (iso)flavone 7- and 3′-O-Glycosyltransferases Can Be Induced by CuCl2
Source: Front Plant Sci. 2021 Feb 9;12:632557. doi: 10.3389/fpls.2021.632557 (PMC7900552; doi:10.3389/fpls.2021.632557)
Supplement: Supplementary file 2 [file Data_Sheet_2.docx]

Supplementary Tables

Table S1 Primers that were used for coloning BcUGTs 2

Table S2 Primers that were used for real time RT-PCR 3

Table S3 Overall quality assessment of raw data from RNA-Seq 4

Table S4 Sequence identities (%) of BcUGTs 5

Table S5 _1_H-NMR (DMSO-*d6*, 500 MHz) and _13_C-NMR (DMSO-*d6*, 125 MHz) data of compound 1b and key HMBC correlations 6

**Table S1 Primers used for cloning BcUGTs.**

| **cCDNA to be amplified** | **Primer name** | **Nucleotide sequence** |
| --- | --- | --- |
| BcUGT1 | BcUGT1-F | G**GAATTC**ATGCAGCTCAAAGAATCAGTAGTAC |
|  | BcUGT1-R | CTAG**TCTAGA**TCAGGCTTCCTTCCTCACAGC |
| BcUGT2 | BcUGT2-F | G**GAATTC**ATGCAGCTCAAAGAATCAGTAGTACTT |
|  | BcUGT2-R | CTAG**TCTAGA**TCAGGCTTCCTTCCTCACAGC |
| BcUGT3 | BcUGT3-F | G**GAATTC**ATGCAGCTCAAAGAATCAGTAGTAC |
|  | BcUGT3-R | GC**TCTAG**ATGTGATTAATGATCAGGCTTCCTTCC |
| BcUGT4 | BcUGT4-F | G**GAATTC**ATGCAGCTCAAGGAATCGGTAG |
|  | BcUGT4-R | GC**TCTAGA**TCAGGCTTCCTTCCTCACAG |
| BcUGT5 | BcUGT5-F | G**GAATTC**ATGCAGCTCAAAGAATCAGTAGTACTT |
|  | BcUGT5-R | GC**TCTAGA**TGTGATTAATGATCAGGCTTCCTTCCT |
| BcUGT6 | BcUGT6-F | G**GAATTC**ACAAGTATGCAGCTGATCAAGGAA |
|  | BcUGT6-R | GC**TCTAGA**TCAAGCTCCCATCCTCAGGGC |
| BcUGT7 | BcUGT7-F | G**GAATTC**ATGCAGCTCAAGGAATCGGTAG |
|  | BcUGT7-R | GC**TCTAGA**TCAAGCTCCCATCCTCAGGGC |
| BcUGT8 | BcUGT8-F | CG**GGATCC**ATGGAGTTCACGCAATCAGTCATC |
| BcUGT9  BcUGT10  BcUGT11 | BcUGT8-R  BcUGT9-F  BcUGT9-R  BcUGT10-F  BcUGT10-R  BcUGT11-F  BcUGT11-R | GC**TCTAGA**AATCAATCAAGCTCCGGTCCC  AA**CTGCAG**ATGGGGCTAAAGGACTCGGTG GC**TCTAGA**TCAAGAATTCCTCAGCGACTTCAC G**GAATTC**AAGAAAAAAATGTCCGCCAAGCTG  GC**TCTAGA**TCAGATTCCACGTTTCATCCTCTCC  AAC**TGCAGA**TGCTCGAATTGGCGAAGCG  GC**TCTAGA**TCAGGCACACAAGTCCTCGA |

Restriction enzyme sites are highlighted by bold and underline format.

**Table S2 Primers used for real time RT-PCR.**

| **cCDNA to be amplified** | **Primer name** | **Nucleotide sequence** |
| --- | --- | --- |
| BcUGT1 | q BcUGT1-F | CGAGCAACGGATGAACAAGGTG |
|  | q BcUGT1-R | ACCTCCTCCGCGCTTACAACG |
| BcUGT2 | q BcUGT2-F | CGGTTCCTCTGGGTGGTGCGG |
|  | q BcUGT2-R | GTGCCCACGACTTTACCACCATT |
| BcUGT3 | q BcUGT3-F | CGTTGTAAGCGCGGAGGAGGT |
|  | q BcUGT3-R | CGCCAATGACGACGAACCATCTTGC |
| BcUGT4 | q BcUGT4-F | ATGCAGCTCAAGGAATCGGTAGTACT |
|  | q BcUGT4-R | GTTCGACGACTACGATGGTGACGG |
| BcUGT5 | q BcUGT5-F | GCCTCCCGTGTTCGCCTTCATT |
|  | q BcUGT5-R | TGAAGTTGCCGTCTAGCTTGGACT |
| BcUGT6 | q BcUGT6-F | CTCCGTCACCATCGTCGTCGT |
|  | q BcUGT6-R | GGGGAGGCGGTGGAAGGAGAT |
| BcUGT7 | q BcUGT7-F | CTCCGTCACCATCGTCGTAGTCG |
|  | q BcUGT7-R | GAGGAGGTGAGGGCATGCGAG |
| BcUGT8 | q BcUGT8-F | GCCACGCCTCCTGTCTACTGC |
|  | q BcUGT8-R | ACCGGCCGTCTCCTTCAGC |
| GADPH | GADPH-F | GGCGATCTCTTTCTCTATCCC |
| Actin | GADPH-R  Actin-F  Actin-R | GCTGGATGCTTCTACCACT  TGCCATGTATGTCGCTATCCAG  GTGCATATCCTTCATAAATTGGAAC |

**Table S3 Overall quality assessment of raw data from RNA-Seq.**

| **Sample** | **Raw Reads** | **Clean Reads** | **Clean Bases** | **Error**  **(%)** | **Q20**  **(%)** | **Q30**  **(%)** | **GC Content (%)** |
| --- | --- | --- | --- | --- | --- | --- | --- |
| BC24_1 | 54292568 | 53312228 | 8G | 0.03 | 0.03 | 93.79 | 93.79 |
| BC24_2 | 62429522 | 61631606 | 9.24G | 0.02 | 0.02 | 95.12 | 95.12 |
| BC24_3 | 53266288 | 51952468 | 7.79G | 0.02 | 0.02 | 95.05 | 95.05 |
| Cu24_1 | 56508716 | 55314082 | 8.3G | 0.03 | 0.03 | 93.42 | 93.42 |
| Cu24_2 | 52080460 | 51337000 | 7.7G | 0.02 | 0.02 | 94.79 | 94.79 |
| Cu24_3 | 65226726 | 63816542 | 9.57G | 0.02 | 0.02 | 94.73 | 94.73 |

**Table S4** **Sequence identities (%) of** **BcUGTs.**

|  | BcUGT1 | BcUGT2 | BcUGT3 | BcUGT4 | BcUGT5 | BcUGT6 | BcUGT7 | BcUGT8 |
| --- | --- | --- | --- | --- | --- | --- | --- | --- |
| BcUGT1 | 100 | 96.1 | 97.9 | 89.0 | 94.0 | 91.2 | 89.4 | 70.2 |
| BcUGT2 | 96.1 | 100 | 94.4 | 92.6 | 93.44 | 89.7 | 92.3 | 71.6 |
| BcUGT3 | 97.3 | 93.8 | 100 | 93.1 | 95.3 | 89.9 | 89.8 | 70.4 |
| BcUGT4 | 89.5 | 90.3 | 91.8 | 100 | 94.5 | 90.3 | 93.0 | 72.7 |
| BcUGT5 | 91.8 | 88.7 | 93.6 | 94.2 | 100 | 92.7 | 90.9 | 71.1 |
| BcUGT6 | 85.9 | 84.1 | 81.7 | 87.9 | 90.6 | 100 | 97.1 | 71.0 |
| BcUGT7 | 84.8 | 87.2 | 86.1 | 91.2 | 89.5 | 96.5 | 100 | 73.2 |
| BcUGT8 | 67.4 | 65.4 | 66.8 | 67.2 | 68.7 | 68.3 | 67.1 | 100 |

cDNAs of BcUGTs are highlighted with a blue background and those of BcUGTs proteins are highlighted with a gray background.

**Table S5 _1_H-NMR (DMSO-*d*6, 500 MHz) and _13_C-NMR (DMSO-*d*6, 125 MHz) data of compound 1b and key HMBC correlations.**

| **Position** | **δ H (m, J in Hz)** | **δ C** | **HMBC** |
| --- | --- | --- | --- |
| **2** | 8.43 (1H, s) | 155.5 | C3, C4, C8a, C1’ |
| **3** |  | 126.5 |  |
| **4** |  | 180.7 |  |
| **4a** |  | 105.3 |  |
| **5** |  | 153.8 |  |
| **6** |  | 132.0 |  |
| **7** |  | 158.1 |  |
| **8** | 6.53 (1H, s) | 94.4 | C4a, C6, C7, C8a |
| **8a** |  | 153.1 |  |
| **1’** |  | 122.6 |  |
| **2’** | 6.96 (1H, d,2) | 110.4 | C3, C3’, C4’, C1’, C6’ |
| **3’** |  | 151.2 |  |
| **4’** |  | 153.1 |  |
| **5’** |  | 138.7 |  |
| **6’** | 6.97 (1H, d,2) | 108.0 | C3, C2’, C4’, C1’, C5’ |
| **6-OCH_3_** | 3. 76 (3H, s) |  | C6 |
| **4’-OCH_3_** | 3.81 (3H, s) |  | C4’ |
| **5’-OCH_3_** | 3.75 (3H, s) |  | C5’ |
| **5 -OH** | 13.01 (br, s) |  | C4a, C5, C6 |
| **7 -OH** | 10.93 (br, s) |  |  |
| **Glc 1”** | 4.91 (1H, d,7.2) | 101.3 | C3’, C3”, C5” |
